# Molecular and Clinical Characterization of CCT2 Expression and Prognosis *via* Large-Scale Transcriptome Profile of Breast Cancer

**DOI:** 10.3389/fonc.2021.614497

**Published:** 2021-04-02

**Authors:** Qiang Liu, Yihang Qi, Xiangyi Kong, Xiangyu Wang, Wenxiang Zhang, Jie Zhai, Yazhe Yang, Yi Fang, Jing Wang

**Affiliations:** ^1^ Department of Breast Surgical Oncology, National Cancer Center/National Clinical Research Center for Cancer/Cancer Hospital, Chinese Academy of Medical Sciences and Peking Union Medical College, Beijing, China; ^2^ Massachusetts General Hospital, Harvard Medical School, Harvard University, Boston, MA, United States

**Keywords:** breast cancer, CCT2 expression, prognostic, molecular chaperone, prognosis

## Abstract

Molecular chaperones play important roles in regulating various cellular processes and malignant transformation. Expression of some subunits of molecular chaperone CCT/TRiC complex have been reported to be correlated with cancer development and patient survival. However, little is known about the expression and prognostic significance of Chaperonin Containing TCP1 Subunit 2 (CCT2). CCT2 is a gene encoding a molecular chaperone that is a member of the chaperonin containing TCP1 complex (CCT), also known as the TCP1 ring complex (TRiC). Through the Cancer Genome Atlas (TCGA) and Molecular Taxonomy of Breast Cancer International Consortium (METABRIC) databases, we systematically reviewed a total of 2,994 cases with transcriptome data and analyzed the functional annotation of CCT2 by Gene ontology and KEGG analysis. Univariate and multivariate survival analysis were performed to investigate the prognostic value of CCT2 in breast cancer. We found CCT2 was significantly upregulated in various tumors. In breast cancer, CCT2 expression was significantly upregulated in HER2-positive (HER2+) group, and more malignant group. In addition, we investigated correlations between CCT2 and other CCT members. Interestingly, almost all CCTs expression were positively correlated with each other, but not CCT6B. Survival analysis suggested that CCT2 overexpression was independently associated with worse prognosis of patients with breast cancer, especially in luminal A subtype. In summary, our results revealed that CCT2 might be involved in regulating cell cycle pathway, and independently predicted worse prognosis in breast cancer patients. These findings may expand understanding of potential anti-CCT2 treatments. To our knowledge, this is the largest and most comprehensive study characterizing the expression pattern of CCT2 together with its prognostic values in breast cancer.

## Introduction

According to the “global cancer statistics” released by the World Health Organization (WHO) in 2015, approximately 1.15 million new cases of breast cancer are diagnosed every year and it accounts for 23% of all female malignancies; there are approximately 410,000 deaths every year, accounting for 14% of deaths due to cancer in women worldwide (1). Although breast cancer is one of the solid tumors of best prognosis and outcome, given that the figure differs significantly among different subtypes, there are still many problems to be solved urgently. With the beginning of the new era of precision medicine, we should give more emphasis on individualized and accurate diagnosis and treatment of breast cancer. Therefore, to seek for novel and promising biomarker for both diagnosis and treatment as well as effective therapeutic target is a major and pressing issue for us.

Although the hardened armors of cancer such as genomic instability, uncontrolled proliferation, metastasis, and so on make it a well-equipped army to fight against our various therapeutics (2, 3), it does have a soft spot: its dependency on major cellular processes like transcription, translation, splicing, protein degradation, and protein-folding (4). During this significant process, proteostasis network (PN), contributing a lot to keep proteome balanced, plays an important role in maintaining native function of proteins and guaranteeing the health of cell and organism. As the central components of the PN, one substance called chaperonin is a key player (5). There are various of proteins participating in proliferation, angiogenesis, survival, and migration, which are vitally essential for tumor formation, progression, and metastasis. To produce these proteins, cancer cells become more highly addicted to molecular chaperones since there are more imbalances caused by overexpression of oncogenes and chromosomal abnormalities (6).

Apart from the HSP90 inhibitors, which were found two decades ago and then abandoned due to incomplete inhibition of HSP90, dose-limited toxicity, and insufficient downregulation of client proteins (7, 8), there is another class of protein-folding complexes named chaperonins in recent years. As a large hetero-oligomeric ATP-dependent complex, this type II chaperonin named CCT is constructed by two stacked back-to-back rings, each creating a place called central chamber to sequester and fold substrate polypeptides that are newly synthesized or misfolded (9–12). CCT is composed of eight paralogous subunits: CCT1-8, also known as CCT α, β, γ, δ, ϵ, ζ, η, θ (13). Approximately 10% of newly synthesized proteins in eukaryotic cells are bound and folded under the assistance of CCT (14), and this figure is observed more in cancer cells. Moreover, the substrates in cancer cells consist of some oncogenic proteins as well as mediators such as STAT3, KRAS, and so on (15–18). Given the evidence that CCT facilitates neoplastic transformation, it is a newly emerging and promising substance that could probably serve as diagnostic marker as well as therapeutic target.

Considering the CCT was a complex that many previous studies focused on, without taking its structure constructed by eight different subunits into account, the importance of a single subunit, for example chaperonin containing TCP1 subunit 2 (CCT2 or CCTβ), was considerably undetermined.

According to several limited published studies, increased expression of CCT2 was observed in various tumor cell lines as compared to normal tissues, including liver, prostate, cholecyst, lung, colorectal, and breast cancers (15, 19–23). In terms of breast cancer, though several studies had illustrated the correspondence between CCT2 expression and the growth of breast cancer cells, there was no comprehensive and detailed conclusion based on clinical data towards different biological, clinical, and molecular characteristics of each distinct subtype (19, 24, 25). Therefore, many unknown factors regarding the expression and prognostic significance of CCT2 in breast cancer must be clarified.

In the present study, we assessed the CCT2 expression status and related biological process by characterizing transcriptome data across two comprehensive genomic databases including a total of 2,994 breast cancer samples. Further, we also explored relationships between CCTs gene family, and their prognostic value. To our best knowledge, this is the largest and most comprehensive study characterizing CCT2 expression in whole grade breast tumor masses.

## Methods and Materials

### Data Acquisition

TCGA dataset on breast invasive carcinoma was downloaded and processed using GDCRNATools (access date: Feb 01, 2020) (26). Raw counts data normalized by TMM implemented in edgeR (27) was then transformed by voom in limma (28), and only genes with cpm > 1 in more than half of the samples were kept. Sieved TCGA breast cancer clinical data was kindly provided by Dr. Hai Hu and Dr. Jianfang Liu in Chan Soon-Shiong Institute of Molecular Medicine at Windber. HER2 status was recalled using DNA copy number for cases without an IHC or FISH status. Standardized survival data from TCGA Pan-Cancer Clinical Data Resource (TCGA-CDR) (29) was utilized in this study. METABRIC dataset (30) on breast cancer (METABRIC, Nature 2012) acquired from cBioPortal (http://www.cbioportal.org/) were utilized for this study (access date: Feb 01, 2020). CCT2 expression data in GSE15852, GSE54002, GSE45827, and GSE42568 datasets were collected from GENT2 database (31) (http://gent2.appex.kr/gent2/), a newly updated platform for exploring gene expression patterns across tumor and normal tissues. Gene expression patterns of CCT2 across tumor and normal tissues were assessed using GENT2 database.

### Kaplan-Meier Plotter Database Analysis

The Kaplan Meier plotter database (32) is capable to assess the effect of 54k genes on survival in 21 cancer types, breast cancer is the largest dataset in Kaplan-Meier plotter containing a total of 6,234 samples. The effect of CCT2 expression on survival together with hazard ratio (HR) with 95% confidence intervals and log-rank P-value in breast cancer was estimated by Kaplan-Meier plotter (http://kmplot.com/analysis).

### TIMER Database Analysis

TIMER database (https://cistrome.shinyapps.io/timer/) is a comprehensive web platform containing 10,897 samples for systematical analysis of immune infiltrates across 32 cancer types from TCGA database (33). The “DiffExp” module was used to explore the differential expression of CCT2 between tumor and adjacent normal tissues, and Wilcoxon test was applied to determine statistical significance of differential expression.

### Functional Enrichment Analysis

GO (34) and Kyoto Encyclopedia of Genes and Genomes (KEGG) pathway enrichment (35) was performed using clusterProfiler package in statistical software R version 3.6.0. (http://www.r-project.org/). GO terms and KEGG pathways with adjusted P-value less than 0.05 were considered to be statistically significant. Dot plot of enriched KEGG pathways were plotted using clusterProfiler package (36).

### Statistical Analyses

Chi-square tests were performed to assess possible associations between CCT2 expression and clinicopathological characteristics. One-way analysis of variance (ANOVA) or T-test was used to determine the differences in CCT2 expression between clinicopathologic characteristics. Survival analysis was estimated using the Kaplan-Meier method, and any differences in survival were evaluated with log-rank test. Univariate and multivariable Cox proportional hazards regression was used to assess association with OS. Gene expression correlation was analyzed by Pearson correlation coefficient. All statistical tests were performed using R software version 3.6.0. P-value <0.05 was considered statistically significant.

## Results

### Expression Pattern of CCT2 in Various Cancers

**Figure 1:**
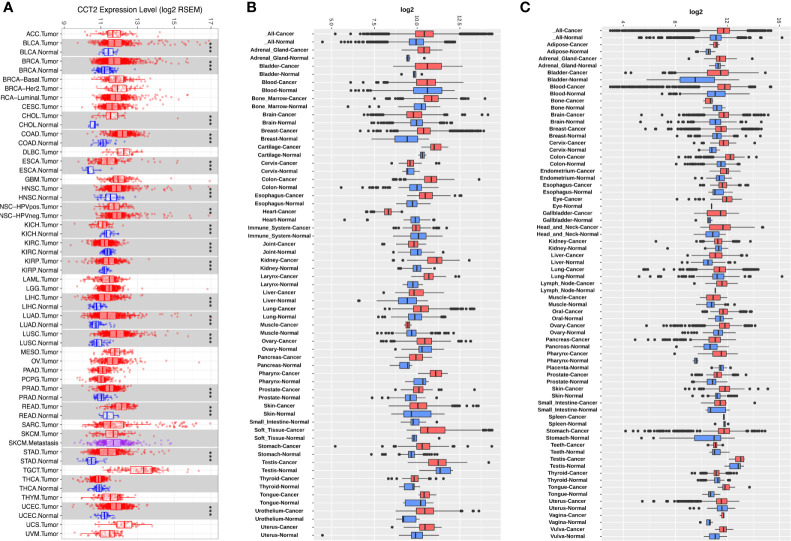
CCT2 expression levels in multiple types of human cancers. **(A)** CCT2 expression levels in all tumors and adjacent normal tissues across TCGA (*P < 0.05, **P <0.01, ***P < 0.001). **(B, C)** Expression levels of CCT2 in 72-paired cancer and normal tissues derived from GPL570 and GPL96 microarray platform.

To determine the mRNA levels of CCT2 in multiple human cancers, we analyzed expression of CCT2 using RNA-sequencing (RNA-seq) data derived from TCGA database. The expression of CCT2 in tumor and adjacent normal tissues across all tumors in TCGA were shown in **Figure 1A**. CCT2 expression was significantly higher in BLCA (bladder urothelial carcinoma), BRCA (breast invasive carcinoma), CHOL (Cholangiocarcinoma), COAD (colon adenocarcinoma), ESCA (Esophageal carcinoma), HNSC (head and neck cancer), KIRP (Kidney renal papillary cell carcinoma), LIHC (liver hepatocellular carcinoma), LUAD (lung adenocarcinoma), LUSC (Lung squamous cell carcinoma), PRAD (Prostate adenocarcinoma), READ (Rectum adenocarcinoma), STAD (stomach adenocarcinoma), and UCEC (Uterine Corpus Endometrial Carcinoma) when compared with adjacent normal tissues. However, CCT2 expression was significantly lower in only two types of cancers, that were, KICH (Kidney Chromophobe) and KIRC (Kidney renal clear cell carcinoma). To validate the expression pattern of CCT2 in various cancers, we further analyzed CCT2 expression in 72-paired tissues across more than 68,000 samples using GENT2 database. Both results from GPL570 and GPL96 microarray platforms revealed that global CCT2 expression was higher in tumor tissues compared with normal tissues (**Figures 1B, C**). CCT2 was higher in most of the tumor tissues when compared with normal tissues. Particularly, the global expression of CCT2 in breast cancer tissues was higher than normal tissues.

### Association Between CCT2 Expression and Clinical Characteristics of Breast Cancer Patients

Expression of CCT2 were dichotomized into low- and high-expression groups using the median as a cut-off value. We analyzed the associations of CCT2 expression and clinical characteristics in both TCGA cohort (n = 1090) and METABRIC cohort (n = 1904), results can be found in **Tables 1** and **2**. We found both two cohorts showed that CCT2 expression was significantly associated with HER2 status. CCT2 expression was associated with American Joint Committee on Cancer (AJCC) stage and age in METABRIC cohort, but not TCGA cohort. CCT2 expression was significantly associated with TNM stage in TCGA cohort, but not ER status. Moreover, CCT2 expression was found to be associated with tumor grade in METABRIC cohort, but not tumor size and ER status.

**Table 1:** Clinicopathologic characteristics according to expression level of *CCT2* mRNA in TCGA database.

		Expression		
	Total (n = 1,090)	CCT2 high (n = 545)	CCT2 low (n = 545)	P-value
**Age (years)**				
>=55	517 (47.4%)	256 (47.0%)	261 (47.9%)	0.808
<55	573 (52.6%)	289 (53.0%)	284 (52.1%)	
**T stage**				
T1	279 (25.6%)	127 (23.3%)	152 (27.9%)	0.012
T2	631 (57.9%)	338 (62.0%)	293 (53.8%)	
T3	137 (12.6%)	56 (10.3%)	81 (14.9%)	
T4	40 (3.7%)	23 (4.2%)	17 (3.1%)	
Unknown	3 (0.3%)	1 (0.2%)	2 (0.4%)	
**N stage**				
N0	514 (47.2%)	247 (45.3%)	267 (49.0%)	0.007
N1	360 (33.0%)	176 (32.3%)	184 (33.8%)	
N2	120 (11.0%)	76 (13.9%)	44 (8.1%)	
N3	76 (7.0%)	31 (5.7%)	45 (8.3%)	
Unknown	20 (1.8%)	15 (2.8%)	5 (0.9%)	
**M stage**				
M0	907 (83.2%)	473 (86.8%)	434 (79.6%)	0.005
M1	22 (2.0%)	10 (1.8%)	12 (2.2%)	
Unknown	161 (14.8%)	62 (11.4%)	99 (18.2%)	
**AJCC stage**				
I	181 (16.6%)	83 (15.2%)	98 (18.0%)	0.429
II	621 (57.0%)	308 (56.5%)	313 (57.4%)	
III	250 (22.9%)	134 (24.6%)	116 (21.3%)	
IV	20 (1.8%)	9 (1.7%)	11 (2.0%)	
Unknown	18 (1.7%)	11 (2.0%)	7 (1.3%)	
**ER status**				
Negative	236 (21.7%)	120 (22.0%)	116 (21.3%)	0.154
Positive	803 (73.7%)	393 (72.1%)	410 (75.2%)	
Unknown	51 (4.7%)	32 (5.9%)	19 (3.5%)	
**PR status**				
Negative	343 (31.5%)	184 (33.8%)	159 (29.2%)	0.081
Positive	694 (63.7%)	330 (60.6%)	364 (66.8%)	
Unknown	53 (4.9%)	31 (5.7%)	22 (4.0%)	
**HER2 status**				
Negative	895 (82.1%)	424 (77.8%)	471 (86.4%)	<0.001
Positive	168 (15.4%)	100 (18.3%)	68 (12.5%)	
Unknown	27 (2.5%)	21 (3.9%)	6 (1.1%)	

**Table 2:** Clinicopathologic characteristics according to expression level of *CCT2* mRNA in METABRIC database.

		Expression		
	Total (n = 1,904)	CCT2 high (n = 952)	CCT2 low (n = 952)	P-value
**Age (years)**				
>=55	952 (50.0%)	514 (54.0%)	438 (46.0%)	<0.001
<55	952 (50.0%)	438 (46.0%)	514 (54.0%)	
**Tumor size**				
>=2 cm	592 (31.1%)	286 (30.0%)	306 (32.1%)	0.33
<2 cm	1292 (67.9%)	657 (69.0%)	635 (66.7%)	
Unknown	20 (1.1%)	9 (0.9%)	11 (1.2%)	
**AJCC stage**				
0	4 (0.2%)	3 (0.3%)	1 (0.1%)	0.003
I	475 (24.9%)	212 (22.3%)	263 (27.6%)	
II	800 (42.0%)	416 (43.7%)	384 (40.3%)	
III	115 (6.0%)	66 (6.9%)	49 (5.1%)	
IV	9 (0.5%)	8 (0.8%)	1 (0.1%)	
Unknown	501 (26.3%)	247 (25.9%)	254 (26.7%)	
**Tumor Grade**				
I	165 (8.7%)	56 (5.9%)	109 (11.4%)	<0.001
II	740 (38.9%)	332 (34.9%)	408 (42.9%)	
III	927 (48.7%)	540 (56.7%)	387 (40.7%)	
Unknown	72 (3.8%)	24 (2.5%)	48 (5.0%)	
**ER status**				
Negative	445 (23.4%)	222 (23.3%)	223 (23.4%)	1
Positive	1459 (76.6%)	730 (76.7%)	729 (76.6%)	
**PR status**				
Negative	895 (47.0%)	466 (48.9%)	429 (45.1%)	0.098
Positive	1,009 (53.0%)	486 (51.1%)	523 (54.9%)	
**HER2 status**				
Negative	1,668 (87.6%)	812 (85.3%)	856 (89.9%)	0.003
Positive	236 (12.4%)	140 (14.7%)	96 (10.1%)	

### CCT2 mRNA Expression Pattern in Breast Cancer

**Figure 2:**
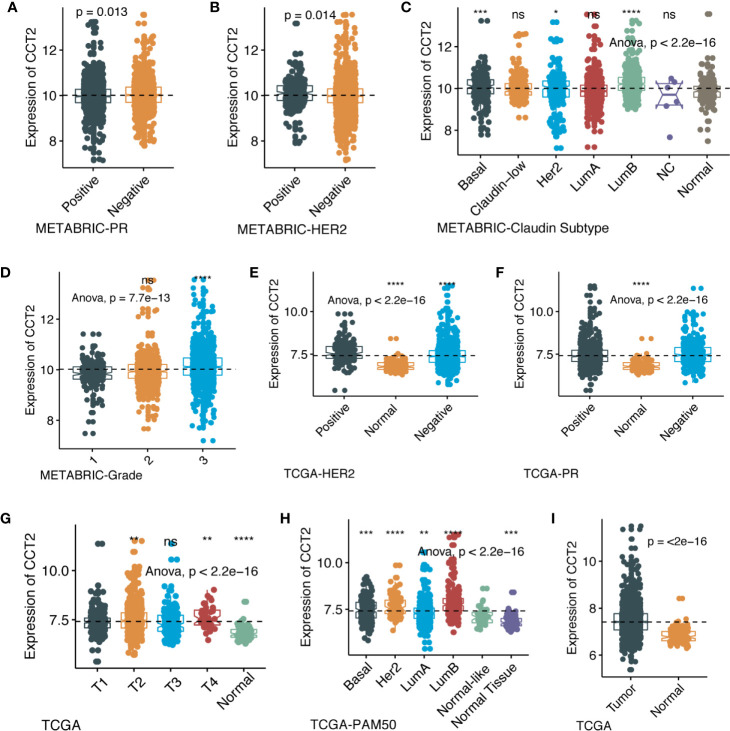
CCT2 expression in different molecular subtypes and stage of transcriptional classification scheme in TCGA and METABRIC cohort. (*P < 0.05, **P < 0.01, ***P < 0.001, ***P < 0.0001), expression pattern of CCT2 in METABRIC database **(A–D)**, expression pattern of CCT2 in TCGA database **(E–I)**. ns, no significance.

**Figure 3:**
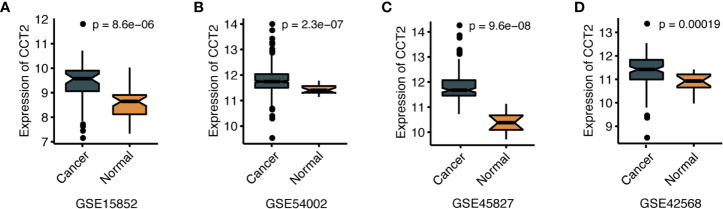
CCT2 expression between cancer and normal tissues in four independent microarray datasets **(A–D)**.

We further explored the differences in CCT2 expression between different clinicopathologic groups. CCT2 expression is significantly higher in PR positive (PR+) group (p = 0.013) and HER2 negative (HER2−) group (p = 0.014) (**Figures 2A, B**), and CCT2 overexpression in HER2+ group was also validated in TCGA cohort, but not PR− group (**Figures 2E, F**). In METABRIC cohort, CCT2 expression was higher in basal, HER2-enriched, luminal B group when compared with normal-like group (**Figure 2C**). CCT2 overexpression was found to be significant in Grade 3 when compared with Grade 1 (P < 0.0001) (**Figure 2D**). In TCGA cohort, elevated expression of CCT2 was found in higher T stage, and more aggressive subtype. CCT2 expression was significantly higher in tumor tissues compared with normal tissues (P < 0.0001) (**Figures 2G–I**), and this result was further validated in four independent microarray datasets derived from GEO database (**Figures 3A–D**).

### Association of CCT2 Expression and Patient Survival in Breast Cancer

**Figure 4:**
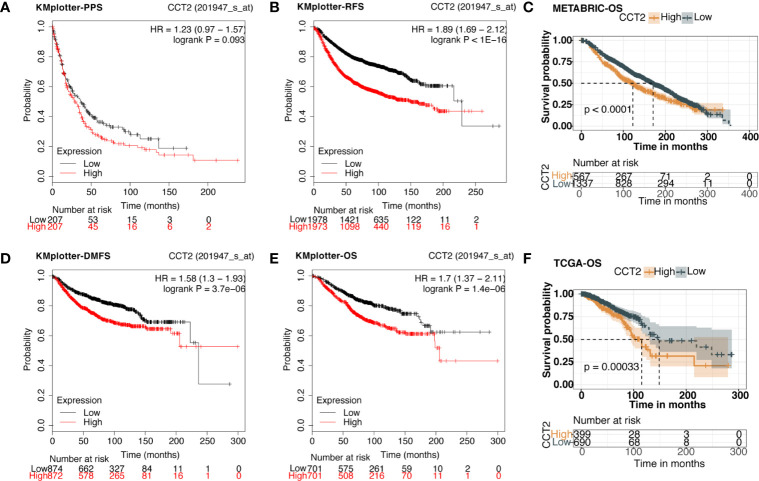
Kaplan-Meier survival curves comparing the high and low expression of CCT2 in breast cancer. **(A, B, D, E)** Survival analysis derived from KMplotter database, and overall survival (OS), relapse-free survival (RFS), postprogression survival (PPS), and distant metastasis-free (DMFS); Hazard ratio, HR. **(C**, **F)** Survival analysis of CCT2 expression using TCGA and METABRIC cohorts.

**Figure 5:**
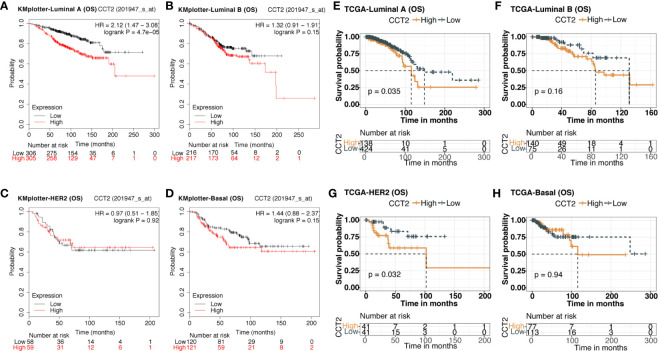
Kaplan-Meier survival curves comparing the high and low expression of CCT2 in breast cancer molecular subtype. **(A–D)** Survival analysis of CCT2 in breast cancer molecular subtype using KMplotter database. **(E–H)** Survival analysis of CCT2 in TCGA.

We explored the prognostic value of CCT2 expression using KM-plotter database containing a total of 6,243 breast cancer samples. Kaplan-Meier analysis revealed that higher CCT2 expression was associated with both worse overall survival (OS), relapse-free survival (RFS), and distant metastasis-free (DMFS) but not postprogression survival (PPS) (**Figures 4A–B, D–E**). CCT2 higher expression significantly correlated with worse OS was further validated in independent METABRIC cohort and TCGA cohort (**Figures 4C, F**). Furthermore, we assessed the prognostic value of CCT2 expression in subtype level, we found that higher expression significantly predicts worse OS in luminal A group in both KMplotter database (P < 0.0001) and TCGA cohort (p < 0.0001) (**Figures 5A, E, G**), but not luminal B, HER2, and basal group (**Figures 5B–D, F, H**).

### Univariate and Multivariate Analyses

**Figure 6:**
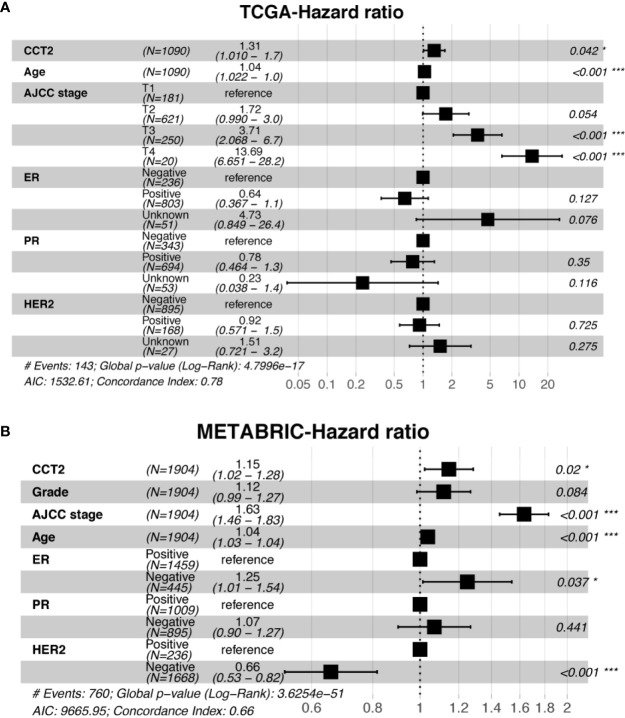
Multivariate analysis of CCT2 expression adjusting for ER, PR, HER2, AJCC stage, age, and stage in TCGA cohorts **(A)** as well as METABRIC cohort **(B)**. *P < 0.05, **P < 0.01, ***P < 0.001.

Expression level of CCT2 mRNA was a significant factor in univariateunivariate analysis of both TCGA (HR, 1.306; 95% CI, 1.037–1.644; p = 0.023) and METABRIC (HR, 1.18; 95% CI, 1.075–1.295; p < 0.001) datasets (**Table 3**). We also found CCT2 was an independent significant prognostic factor for breast cancer according to multivariate analysis of TCGA cohort after adjusting for age, AJCC stage, ER status, PR status, as well as HER2 status (**Figure 6A**). Interestingly, CCT2 expression was also an independent prognostic factor for breast cancer in multivariate analysis of METABRIC cohort after adjusting for age, AJCC stage, Grade, ER status, PR status, as well as HER2 status (**Figure 6B**).

**Table 3:** Univariable analyses using TCGA and METABRIC databases.

Factors	Univariable analysis (TCGA)		Factors	Univariable analysis (METABRIC)	
	HR (95% CI)	P		HR (95% CI)	P
**Age**	1.032 (1.020–1.045)	<0.001	**Age**	1.036 (1.030–1.041)	<0.001
**AJCC stage**	2.207 (1.764–2.762)	<0.001	**AJCC stage**	1.813 (1.622–2.027)	<0.001
**ER**			**ER**		
Positive	Reference		Positive	Reference	
Negative	1.389 (0.964–2.003)	0.078	Negative	1.180 (1.024–1.358)	0.022
Unknown	2.746 (1.425–5.292)	0.003	Unknown	—	
**PR**			**PR**		
Positive	Reference		Positive	Reference	
Negative	1.347 (0.961–1.888)	0.083	Negative	1.269 (1.128–1.429)	<0.001
Unknown	2.065 (1.036–4.119)	0.040	Unknown	—	
**HER2**			**HER2**		
Positive	Reference		Positive	Reference	
Negative	0.893 (0.566–1.409)	0.626	Negative	0.688 (0.578–0.818)	<0.001
Unknown	2.332 (1.149–4.730)	0.019	Unknown	—	
**CCT2**	1.306 (1.037–1.644)	0.023	**CCT2**	1.180 (1.075–1.295)	<0.001
**Grade**	—		**Grade**	1.273 (1.156–1.403)	<0.001

### CCT2-Related Signaling Pathways Identified Using Functional Enrichment Analysis

**Figure 7:**
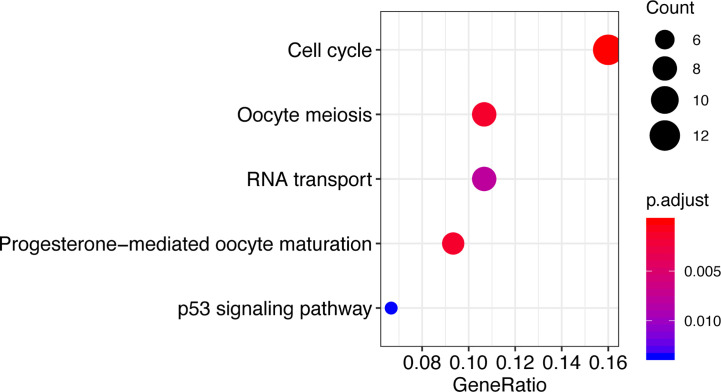
Functional enrichment analysis shows KEGG enriched pathways of CCT2-related genes.

To explore the potential functional role of CCT2, genes correlated with CCT2 expression (Pearson |R|>=0.4) were screened out (n = 140) (**Table S1**), these genes were further used to do functional enrichment analysis in R using cluster Profiler package (35). Interestingly, GO analysis revealed that these genes were mainly involved in protein folding and binding biological processes (**Table S2**). KEGG enrichment analysis revealed that these genes were significantly enriched in cell cycle, oocyte meiosis, progesterone-mediated oocyte maturation, and RNA transport as well as p53 signaling pathway (**Figure 7**).

### Correlations Between CCTs Gene Family and Prognostic Value

**Figure 8:**
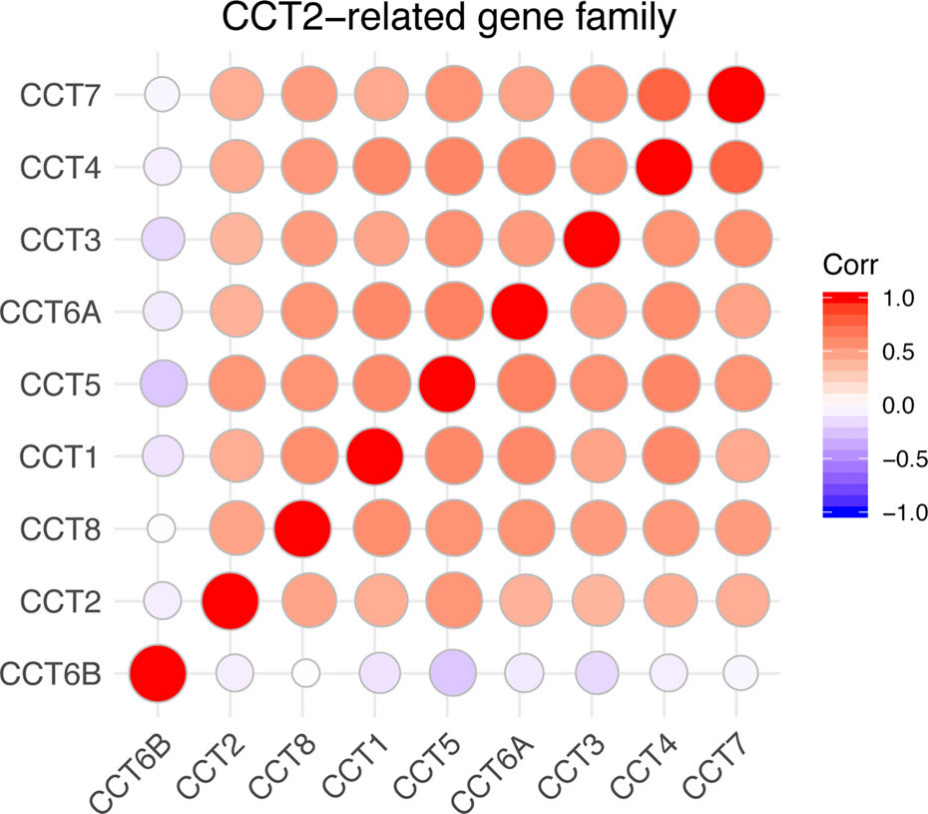
Correlations between CCTs gene family. Corr denotes Pearson correlation coefficient. P-value <0.05 was considered statistically significant. No significant correlations were shown in blank.

We calculated the correlations of CCTs with each other by analyzing their mRNA expressions in TCGA cohort. Interestingly, we found almost all CCT genes were significantly positively correlated with each other, including CCT1, CCT2, CCT3, CCT4, CCT5, CCT6A, and CCT7 as well as CCT8, but not CCT6B (**Figure 8**). Furthermore, we systematically assessed the prognostic value of CCTs gene family using univariate analyses in both TCGA and METABRIC cohort (**Table 4**). CCT4 expression in METABRIC dataset can’t be accessed thereby prognostic value in METABRIC cohort was unknown. In summary, only CCT2 and CCT5 were significantly correlated with OS in both two cohorts.

**Table 4:** Univariate analysis of CCTs in TCGA and METABRIC cohort.

Varname	Univariable analysis (TCGA)		Varname	Univariable analysis (METABRIC)	
	HR (95%CI)	P		HR (95%CI)	P
**CCT1**	1.72 (1.34–2.21)	P<0.001	**CCT1**	1.04 (0.96–1.14)	0.344
**CCT2**	1.31 (1.04–1.64)	0.023	**CCT2**	1.18 (1.07–1.30)	0.001
**CCT3**	1.20 (0.92–1.57)	0.173	**CCT3**	1.07 (0.95–1.20)	0.293
**CCT4**	1.53 (1.14–2.05)	0.004	**CCT4**	—	—
**CCT5**	1.30 (1.04–1.64)	0.023	**CCT5**	1.27 (1.13–1.42)	P<0.001
**CCT6A**	1.15 (0.91–1.46)	0.242	**CCT6A**	1.07 (0.95–1.21)	0.257
**CCT6B**	0.97 (0.8–1.18)	0.776	**CCT6B**	0.77 (0.67–0.88)	P<0.001
**CCT7**	1.38 (0.99–1.92)	0.055	**CCT7**	1.04 (0.96–1.13)	0.327
**CCT8**	1.37 (1.03–1.84)	0.032	**CCT8**	0.92 (0.82–1.02)	0.124

## Discussion

Our work revealed that CCT2 tends to be overexpressed in tumor tissues compared with normal tissues. Moreover, CCT2 was overexpressed in more malignant grades and molecular subtypes of breast cancer. Genes correlated with CCT2 expression were mainly enriched in cell cycle pathway and also P53 signaling pathway. To the clinical aspects, our results indicated CCT2 expression was independently associated with worse prognosis of patients with breast cancer, especially in luminal A subtype. Additionally, we also explored potential relationships between CCTs gene family and their prognostic role in breast cancer.

Many previous studies have focused on colorectal cancer, gallbladder cancer, liver cancer, prostate cancer, small cell lung cancer, and so on. For example, Park et al. found that the tissues of human colorectal cancer showed greater CCT2 expression than did the normal colon tissues, which indicated that higher CCT2 expression in tumor tissues from colorectal cancer patients reduced their survival rate. Besides, on the basis of the research conducted by Zou et al., in gallbladder cancer, the positive expression of PDIA3 and CCT2 was significantly associated with clinicopathological features of both squamous carcinoma/adenosquamous carcinoma and adenocarcinoma specimens, consisting of lymph node metastasis and high TNM stage (22). Though there were several valuable outcomes, much more work related to BLCA, ESCA, HNSC, STAD, UCEC, and renal tumors remains to be done, which will inevitably lead to a much more comprehensive understanding of the function of CCT2 in numerous cancers.

With regard to breast cancer, there were some published researches concerning CCT2 of high-quality. The first one was a study conducted by AH Charpentier et al. released in 2000, they illustrated that Pescadillo and chaperonin CCT2 were two presumptive autocrine/paracrine factors of potential function in the regulation of the growth of breast cancer cells, which were identified to be highly upregulated by E2 (17beta estradiol) (24). Besides, the research conducted by Stephen T. Guest et al. represented some unique new findings. They identified that CCT1 and CCT2 were necessary for growth/survival of breast cancer cells *in vitro* and were determinants of overall survival in breast cancer patients (19). Apart from that, another research conducted by Anne E. Showalter et al., published in this year also drew some conclusions. By depleting or overexpressing the subunit in breast cancer and breast epithelial cells, they found that increasing CCT2 in cells by 1.3–1.8-fold also increased other CCT subunits’ (CCT3, CCT4, and CCT5) levels, while silencing the expression of CCT2 by ~50% was able to cause other CCT subunits to reduce. Besides, their study also represented that cells expressing higher CCT2 were more invasive and showed a higher proliferative index, and depletion of CCT2 in a syngeneic murine model of triple negative breast cancer (TNBC) had a potential to prevent tumor growth (25).

Though all these previous studies laid emphasis on the significance of CCT2 in breast cancer, what they focused on was only the growth and survival of breast cancer cells. There was no comprehensive and detailed conclusion towards different biological, clinical, and molecular characteristics of each distinct subtype. More importantly, transcriptome data we used in this study were derived from the top two biggest independent breast cancer databases, which enabled our outcomes much more overall and reliable.

As for other functions of CCT2, Park et al. found that reduction in CCT2 inhibited tumor induction by Gli-1, and ubiquitination-mediated Gli-1 degradation by β-TrCP occurred during incomplete folding of Gli-1 in hypoxia. CCT2 correlates with Gli-1 expression is an important determinant of survival in the colorectal cancer patients. Besides, based on the study conducted by Lu et al., they discovered that as an essential enzyme in *de novo* synthesis of purine, phosphoribosylformylglycinamidine synthase (PFAS) interacted with several proteins which played physiological roles in tumor development including CAD, CCT2, PRDX1, and PHGDH, and it was also able to deamidate PHGDH, and induce other posttranslational modification into CAD, CCT2, and PRDX1 (37). When it comes to other subunits of CCT complex, previous studies have reported some valuable points. In various cancers, the expression levels of different CCT subunits were upregulated in varying degrees: CCT3 in hepatocellular carcinoma (38), and CCT8 in hepatocellular carcinoma and glioblastoma (39, 40). Based on study conducted by Hallal et al., extracellular vesicles from neurosurgical aspirates identified CCT6A as a potential glioblastoma biomarker with prognostic significance (41). Another group found that overexpression of CCT1 in yeast did not exert any effect on levels of assembled complex, but the CCT1 subunits which were remained soluble in the cytosol had inherent activity of protein-folding (42). In terms of CCT subunits acting as monomers, scientists found that CCT4 was able to produce a protrusion phenotype by interacting with microtubules and p150glued (43, 44). CCT5 and CCT8 could colocalize with actin fibers outside of the oligomer54, and CCT5 also played a key role in the transcriptional regulation of actin (45). Previous study also represented CCT5 had correspondence with breast cancer. Ooe A et al. discovered that CCT5, RGS3, and YKT6 mRNA expressions, which were upregulated in p53-mutated breast cancers, might be involved in resistance to docetaxel and clinically feasible in distinguish the subset of breast cancer patients who may or may not be benefit from docetaxel therapy (46). Apart from that, CCT5 was identified to be closely related to lung cancer. Gao H et al. showed that CCT5 could induce an autoantibody response in non-small cell lung cancer (NSCLC) sera and showed higher expression in NSCLC tissues by Western blot and immunohistochemistry (47). Knockdown of CCT5, PIP4K2A, EXO1, CMBL, OPN3, and KMO, genes within 200 kb up/downstream of the three SNPs that were corresponded with small cell lung cancer (SCLC) overall survival (48). In addition, CCT5 also participated in replication of hepatitis C virus genome through interaction with the viral NS5B protein (49). However, the role of CCT in many diseases, including cancer, is far from fully characterized, needing much more researches and studies towards that.

Consistent to our results, some studies also reported the potential role of inhibiting cancer cell by targeting CCTs. For instance, Showalter Anne E et al., discovered one CCT inhibitor named CT20p, which had access to kill cancer cells in a CCT-dependent manner. In cancer cells where the CCT was inhibited, they were resistant to CT20p killing, while cells where the expression of CCT was increased were susceptible (15, 23). However, given the fact that the complexity of CCT and its multiple subunits, as well as the lack of a complete understanding of CCT substrate selectivity *in vivo*, there are inevitably some challenges that impede the development of feasible and effective therapeutics like CT20p (25). In summary, we discussed the role of CCT2 in tumors together with current researches regarding CCTs gene family. Future research focus on investigating the underlying molecular mechanisms of CCT2 in promoting cancer might yield novel insights for possible treatments by targeting CCT2.

## Data Availability Statement

The datasets presented in this study can be found in online repositories. The names of the repository/repositories and accession number(s) can be found in the article/**Supplementary Material**.

## Author Contributions

QL and YQ carried out the primary literature search. QL performed the data analysis. QL and YQ drafted the manuscript. QL, XW, WZ, JZ, YZY and XK performed the literature search and revised the manuscript. QL, YQ, XK, YF, and JW discussed, revised, and edited the manuscript. All authors contributed to the article and approved the submitted version.

## Funding

This paper was partially sponsored by grants from the National Natural Science Foundation of China, No. 81872160 (JW).

## Conflict of Interest

The authors declare that the research was conducted in the absence of any commercial or financial relationships that could be construed as a potential conflict of interest.
