# Supplementary material for: Molecular and Clinical Characterization of CCT2 Expression and Prognosis via Large-Scale Transcriptome Profile of Breast Cancer
Source: Front Oncol. 2021 Apr 2;11:614497. doi: 10.3389/fonc.2021.614497 (PMC8050343; doi:10.3389/fonc.2021.614497)
Supplement: Supplementary file 1 [file Table_1.docx]

Table S1  Genes correlated with CCT2

| No. | symbol | correlation | pvalue |  |
| --- | --- | --- | --- | --- |
| 1 | CCT2 | 1 | 0 |  |
| 2 | YEATS4 | 0.753926586 | 9.71E-201 |  |
| 3 | NUP107 | 0.718765323 | 6.00E-174 |  |
| 4 | CPSF6 | 0.646981346 | 2.84E-130 |  |
| 5 | CAND1 | 0.635096254 | 3.74E-124 |  |
| 6 | PTGES3 | 0.617355414 | 1.67E-115 |  |
| 7 | LLPH | 0.572820974 | 4.68E-96 |  |
| 8 | XPOT | 0.571732369 | 1.28E-95 |  |
| 9 | HSPD1 | 0.547460881 | 2.98E-86 |  |
| 10 | CCT5 | 0.535449227 | 6.79E-82 |  |
| 11 | UBE2N | 0.52592364 | 1.46E-78 |  |
| 12 | CSE1L | 0.522797432 | 1.72E-77 |  |
| 13 | RAN | 0.517950612 | 7.48E-76 |  |
| 14 | ATP5F1B | 0.513727551 | 1.91E-74 |  |
| 15 | MDM2 | 0.511956533 | 7.31E-74 |  |
| 16 | RACGAP1 | 0.500027317 | 5.11E-70 |  |
| 17 | RAB21 | 0.496294785 | 7.58E-69 |  |
| 18 | PAICS | 0.49050737 | 4.66E-67 |  |
| 19 | DCAF13 | 0.48895805 | 1.38E-66 |  |
| 20 | HSP90AA1 | 0.483594119 | 5.76E-65 |  |
| 21 | PA2G4 | 0.482946147 | 8.99E-65 |  |
| 22 | HSPA9 | 0.482586659 | 1.15E-64 |  |
| 23 | MAD2L1 | 0.482080849 | 1.63E-64 |  |
| 24 | PPAT | 0.481917722 | 1.82E-64 |  |
| 25 | NOL11 | 0.478477783 | 1.90E-63 |  |
| 26 | DENR | 0.476927577 | 5.40E-63 |  |
| 27 | MRPL13 | 0.476781415 | 5.96E-63 |  |
| 28 | CCT8 | 0.473563226 | 5.15E-62 |  |
| 29 | TUBA1B | 0.473427476 | 5.64E-62 |  |
| 30 | KPNA2 | 0.468067916 | 1.94E-60 |  |
| 31 | HSP90AB1 | 0.467207357 | 3.40E-60 |  |
| 32 | CCNB1 | 0.46559539 | 9.71E-60 |  |
| 33 | PRIM1 | 0.463889292 | 2.93E-59 |  |
| 34 | RAB3IP | 0.463871258 | 2.96E-59 |  |
| 35 | PSMD12 | 0.462877328 | 5.62E-59 |  |
| 36 | PPIL1 | 0.462803915 | 5.89E-59 |  |
| 37 | PRELID3B | 0.461775933 | 1.14E-58 |  |
| 38 | STIP1 | 0.460049315 | 3.43E-58 |  |
| 39 | DSCC1 | 0.459269262 | 5.64E-58 |  |
| 40 | RPAP3 | 0.458965387 | 6.84E-58 |  |
| 41 | POLR2K | 0.454972163 | 8.49E-57 |  |
| 42 | RAE1 | 0.454647882 | 1.04E-56 |  |
| 43 | VDAC1 | 0.453909075 | 1.65E-56 |  |
| 44 | MTFR1 | 0.453546813 | 2.07E-56 |  |
| 45 | UBE2V2 | 0.453509066 | 2.12E-56 |  |
| 46 | AURKA | 0.453031152 | 2.85E-56 |  |
| 47 | TIMELESS | 0.452970302 | 2.96E-56 |  |
| 48 | MRPS23 | 0.451795456 | 6.14E-56 |  |
| 49 | TBC1D15 | 0.451393217 | 7.88E-56 |  |
| 50 | AZIN1 | 0.450462729 | 1.40E-55 |  |
| 51 | MRPS35 | 0.449647219 | 2.32E-55 |  |
| 52 | ELOC | 0.449358896 | 2.76E-55 |  |
| 53 | MRPL42 | 0.44780068 | 7.19E-55 |  |
| 54 | VBP1 | 0.446428213 | 1.66E-54 |  |
| 55 | NUDCD1 | 0.444355942 | 5.84E-54 |  |
| 56 | ENOPH1 | 0.444294241 | 6.06E-54 |  |
| 57 | ARMC1 | 0.443671277 | 8.83E-54 |  |
| 58 | FKBP4 | 0.44212752 | 2.24E-53 |  |
| 59 | CDK4 | 0.441537421 | 3.18E-53 |  |
| 60 | TUBA1C | 0.441381678 | 3.50E-53 |  |
| 61 | SLC35E3 | 0.439812393 | 8.92E-53 |  |
| 62 | FRS2 | 0.439265898 | 1.23E-52 |  |
| 63 | GID8 | 0.439132549 | 1.34E-52 |  |
| 64 | PSMD10 | 0.438469374 | 1.98E-52 |  |
| 65 | TBK1 | 0.43749992 | 3.52E-52 |  |
| 66 | KANSL2 | 0.437479331 | 3.56E-52 |  |
| 67 | TMEM70 | 0.43655232 | 6.15E-52 |  |
| 68 | UTP23 | 0.436479364 | 6.42E-52 |  |
| 69 | FBXO45 | 0.436094296 | 8.06E-52 |  |
| 70 | CS | 0.436093136 | 8.06E-52 |  |
| 71 | TMED2 | 0.435740111 | 9.92E-52 |  |
| 72 | PNO1 | 0.434730208 | 1.79E-51 |  |
| 73 | YWHAZ | 0.433695661 | 3.28E-51 |  |
| 74 | BRIX1 | 0.432969672 | 5.01E-51 |  |
| 75 | HSPH1 | 0.432192958 | 7.87E-51 |  |
| 76 | SET | 0.431497574 | 1.18E-50 |  |
| 77 | METAP2 | 0.430736353 | 1.83E-50 |  |
| 78 | CDC6 | 0.428064831 | 8.51E-50 |  |
| 79 | CCT4 | 0.427498255 | 1.18E-49 |  |
| 80 | CDK1 | 0.427105205 | 1.47E-49 |  |
| 81 | PSMD14 | 0.426824725 | 1.73E-49 |  |
| 82 | TOMM70 | 0.426340292 | 2.28E-49 |  |
| 83 | DERL1 | 0.424927456 | 5.08E-49 |  |
| 84 | GLO1 | 0.424617024 | 6.06E-49 |  |
| 85 | CCT7 | 0.423764989 | 9.80E-49 |  |
| 86 | SQLE | 0.422524152 | 1.97E-48 |  |
| 87 | TIMM17A | 0.422393012 | 2.12E-48 |  |
| 88 | RAD21 | 0.421867502 | 2.85E-48 |  |
| 89 | STRAP | 0.420631252 | 5.69E-48 |  |
| 90 | ARL6IP1 | 0.42050142 | 6.12E-48 |  |
| 91 | TMEM97 | 0.420436189 | 6.35E-48 |  |
| 92 | CBX7 | -0.420430938 | 6.36E-48 |  |
| 93 | TCP1 | 0.420401477 | 6.47E-48 |  |
| 94 | MTCH2 | 0.42018327 | 7.31E-48 |  |
| 95 | PSMA6 | 0.419938958 | 8.37E-48 |  |
| 96 | METTL2A | 0.419151016 | 1.30E-47 |  |
| 97 | HMMR | 0.417367029 | 3.49E-47 |  |
| 98 | EIF2S2 | 0.417082764 | 4.08E-47 |  |
| 99 | NCAPG | 0.416998219 | 4.27E-47 |  |
| 100 | NPM1 | 0.41600451 | 7.39E-47 |  |
| 101 | BIRC5 | 0.415570783 | 9.37E-47 |  |
| 102 | H2AFZ | 0.414268334 | 1.91E-46 |  |
| 103 | DNAJA1 | 0.413376504 | 3.11E-46 |  |
| 104 | MTHFD2 | 0.412889248 | 4.06E-46 |  |
| 105 | SRSF1 | 0.412418856 | 5.24E-46 |  |
| 106 | PARPBP | 0.410793251 | 1.26E-45 |  |
| 107 | RPN2 | 0.410766168 | 1.28E-45 |  |
| 108 | METTL1 | 0.410206187 | 1.73E-45 |  |
| 109 | DKC1 | 0.409607388 | 2.39E-45 |  |
| 110 | PGK1 | 0.409521378 | 2.51E-45 |  |
| 111 | KIF18A | 0.408327445 | 4.76E-45 |  |
| 112 | TOP1 | 0.408292482 | 4.85E-45 |  |
| 113 | TXNRD1 | 0.407577448 | 7.11E-45 |  |
| 114 | ATAD2 | 0.406912517 | 1.01E-44 |  |
| 115 | UTP18 | 0.40673935 | 1.11E-44 |  |
| 116 | LEMD3 | 0.406727701 | 1.12E-44 |  |
| 117 | MCM4 | 0.406160637 | 1.51E-44 |  |
| 118 | SPAG5 | 0.405636204 | 2.00E-44 |  |
| 119 | MARS | 0.405447802 | 2.21E-44 |  |
| 120 | CACYBP | 0.40511127 | 2.64E-44 |  |
| 121 | GINS1 | 0.405062601 | 2.71E-44 |  |
| 122 | BTF3L4 | 0.40471043 | 3.26E-44 |  |
| 123 | ATXN7L3B | 0.404677414 | 3.32E-44 |  |
| 124 | TATDN1 | 0.404626168 | 3.41E-44 |  |
| 125 | CCT6A | 0.404501254 | 3.65E-44 |  |
| 126 | LMNB1 | 0.404385962 | 3.88E-44 |  |
| 127 | RARS | 0.403199579 | 7.24E-44 |  |
| 128 | CDC25C | 0.403068023 | 7.76E-44 |  |
| 129 | LRPPRC | 0.402988166 | 8.10E-44 |  |
| 130 | MAPRE1 | 0.402300841 | 1.16E-43 |  |
| 131 | ACTL6A | 0.401853205 | 1.47E-43 |  |
| 132 | SRP54 | 0.401606202 | 1.67E-43 |  |
| 133 | HSPE1 | 0.401344645 | 1.92E-43 |  |
| 134 | PAIP1 | 0.401179035 | 2.09E-43 |  |
| 135 | YWHAB | 0.400691427 | 2.70E-43 |  |
| 136 | PRKAG1 | 0.400510186 | 2.96E-43 |  |
| 137 | CCNE2 | 0.400414158 | 3.12E-43 |  |
| 138 | PCMT1 | 0.400409151 | 3.12E-43 |  |
| 139 | TARS | 0.4002643 | 3.37E-43 |  |
| 140 | EIF3J | 0.40022129 | 3.45E-43 |  |
| 141 | NOLC1 | 0.400089396 | 3.69E-43 |  |
