# Supplementary material for: Molecular and Clinical Characterization of CCT2 Expression and Prognosis via Large-Scale Transcriptome Profile of Breast Cancer
Source: Front Oncol. 2021 Apr 2;11:614497. doi: 10.3389/fonc.2021.614497 (PMC8050343; doi:10.3389/fonc.2021.614497)
Supplement: Supplementary file 2 [file Table_2.docx]

Table S2  GO enrichment analysis

|  | ONTOLOGY | ID | Description | GeneRatio | BgRatio | pvalue | p.adjust | qvalue | geneID | Count |
| --- | --- | --- | --- | --- | --- | --- | --- | --- | --- | --- |
| GO:0006457 | BP | GO:0006457 | protein folding | 22/138 | 280/19623 | 4.50E-17 | 9.04E-14 | 7.00E-14 | CCT2/PTGES3/HSPD1/CCT5/HSP90AA1/HSPA9/CCT8/HSP90AB1/PPIL1/STIP1/RPAP3/VBP1/FKBP4/HSPH1/CCT4/TOMM70/DERL1/CCT7/TCP1/DNAJA1/CCT6A/HSPE1 | 22 |
| GO:0070203 | BP | GO:0070203 | regulation of establishment of protein localization to telomere | 8/138 | 10/19623 | 2.17E-16 | 2.18E-13 | 1.68E-13 | CCT2/CCT5/CCT8/CCT4/CCT7/TCP1/DKC1/CCT6A | 8 |
| GO:0070202 | BP | GO:0070202 | regulation of establishment of protein localization to chromosome | 8/138 | 11/19623 | 7.90E-16 | 3.97E-13 | 3.07E-13 | CCT2/CCT5/CCT8/CCT4/CCT7/TCP1/DKC1/CCT6A | 8 |
| GO:1904816 | BP | GO:1904816 | positive regulation of protein localization to chromosome, telomeric region | 8/138 | 11/19623 | 7.90E-16 | 3.97E-13 | 3.07E-13 | CCT2/CCT5/CCT8/CCT4/CCT7/TCP1/DKC1/CCT6A | 8 |
| GO:1904814 | BP | GO:1904814 | regulation of protein localization to chromosome, telomeric region | 8/138 | 13/19623 | 6.09E-15 | 2.45E-12 | 1.89E-12 | CCT2/CCT5/CCT8/CCT4/CCT7/TCP1/DKC1/CCT6A | 8 |
| GO:0006458 | BP | GO:0006458 | de novo' protein folding | 12/138 | 65/19623 | 2.64E-14 | 8.63E-12 | 6.68E-12 | CCT2/PTGES3/HSPD1/CCT5/HSPA9/CCT8/HSPH1/CCT4/CCT7/TCP1/CCT6A/HSPE1 | 12 |
| GO:0070200 | BP | GO:0070200 | establishment of protein localization to telomere | 8/138 | 15/19623 | 3.01E-14 | 8.63E-12 | 6.68E-12 | CCT2/CCT5/CCT8/CCT4/CCT7/TCP1/DKC1/CCT6A | 8 |
| GO:1904874 | BP | GO:1904874 | positive regulation of telomerase RNA localization to Cajal body | 8/138 | 16/19623 | 5.98E-14 | 1.50E-11 | 1.16E-11 | CCT2/CCT5/CCT8/CCT4/CCT7/TCP1/DKC1/CCT6A | 8 |
| GO:0061077 | BP | GO:0061077 | chaperone-mediated protein folding | 13/138 | 92/19623 | 8.05E-14 | 1.80E-11 | 1.39E-11 | CCT2/PTGES3/HSPD1/CCT5/HSPA9/CCT8/FKBP4/HSPH1/CCT4/CCT7/TCP1/CCT6A/HSPE1 | 13 |
| GO:2000573 | BP | GO:2000573 | positive regulation of DNA biosynthetic process | 12/138 | 75/19623 | 1.61E-13 | 3.24E-11 | 2.51E-11 | CCT2/PTGES3/CCT5/HSP90AA1/CCT8/HSP90AB1/DSCC1/CCT4/CCT7/TCP1/DKC1/CCT6A | 12 |
| GO:1990173 | BP | GO:1990173 | protein localization to nucleoplasm | 7/138 | 11/19623 | 2.35E-13 | 4.30E-11 | 3.32E-11 | CCT2/CCT5/CCT8/CCT4/CCT7/TCP1/CCT6A | 7 |
| GO:1904872 | BP | GO:1904872 | regulation of telomerase RNA localization to Cajal body | 8/138 | 20/19623 | 5.72E-13 | 9.57E-11 | 7.41E-11 | CCT2/CCT5/CCT8/CCT4/CCT7/TCP1/DKC1/CCT6A | 8 |
| GO:0090670 | BP | GO:0090670 | RNA localization to Cajal body | 8/138 | 21/19623 | 9.18E-13 | 1.15E-10 | 8.92E-11 | CCT2/CCT5/CCT8/CCT4/CCT7/TCP1/DKC1/CCT6A | 8 |
| GO:0090671 | BP | GO:0090671 | telomerase RNA localization to Cajal body | 8/138 | 21/19623 | 9.18E-13 | 1.15E-10 | 8.92E-11 | CCT2/CCT5/CCT8/CCT4/CCT7/TCP1/DKC1/CCT6A | 8 |
| GO:0090672 | BP | GO:0090672 | telomerase RNA localization | 8/138 | 21/19623 | 9.18E-13 | 1.15E-10 | 8.92E-11 | CCT2/CCT5/CCT8/CCT4/CCT7/TCP1/DKC1/CCT6A | 8 |
| GO:0090685 | BP | GO:0090685 | RNA localization to nucleus | 8/138 | 21/19623 | 9.18E-13 | 1.15E-10 | 8.92E-11 | CCT2/CCT5/CCT8/CCT4/CCT7/TCP1/DKC1/CCT6A | 8 |
| GO:0051054 | BP | GO:0051054 | positive regulation of DNA metabolic process | 17/138 | 243/19623 | 1.37E-12 | 1.62E-10 | 1.26E-10 | CCT2/PTGES3/CCT5/UBE2N/HSP90AA1/CCT8/HSP90AB1/DSCC1/UBE2V2/TIMELESS/CCT4/CDK1/CCT7/TCP1/DKC1/CACYBP/CCT6A | 17 |
| GO:0007004 | BP | GO:0007004 | telomere maintenance via telomerase | 11/138 | 71/19623 | 2.48E-12 | 2.77E-10 | 2.14E-10 | CCT2/PTGES3/CCT5/HSP90AA1/CCT8/HSP90AB1/CCT4/CCT7/TCP1/DKC1/CCT6A | 11 |
| GO:0051052 | BP | GO:0051052 | regulation of DNA metabolic process | 21/138 | 430/19623 | 2.85E-12 | 3.01E-10 | 2.33E-10 | CCT2/PTGES3/CCT5/UBE2N/HSP90AA1/CCT8/KPNA2/HSP90AB1/DSCC1/UBE2V2/TIMELESS/CDC6/CCT4/CDK1/CCT7/TCP1/NPM1/PARPBP/DKC1/CACYBP/CCT6A | 21 |
| GO:0070199 | BP | GO:0070199 | establishment of protein localization to chromosome | 8/138 | 24/19623 | 3.26E-12 | 3.26E-10 | 2.52E-10 | CCT2/CCT5/CCT8/CCT4/CCT7/TCP1/DKC1/CCT6A | 8 |
| GO:0006278 | BP | GO:0006278 | RNA-dependent DNA biosynthetic process | 11/138 | 73/19623 | 3.41E-12 | 3.26E-10 | 2.52E-10 | CCT2/PTGES3/CCT5/HSP90AA1/CCT8/HSP90AB1/CCT4/CCT7/TCP1/DKC1/CCT6A | 11 |
| GO:0070198 | BP | GO:0070198 | protein localization to chromosome, telomeric region | 8/138 | 28/19623 | 1.35E-11 | 1.23E-09 | 9.51E-10 | CCT2/CCT5/CCT8/CCT4/CCT7/TCP1/DKC1/CCT6A | 8 |
| GO:0010833 | BP | GO:0010833 | telomere maintenance via telomere lengthening | 11/138 | 83/19623 | 1.46E-11 | 1.27E-09 | 9.85E-10 | CCT2/PTGES3/CCT5/HSP90AA1/CCT8/HSP90AB1/CCT4/CCT7/TCP1/DKC1/CCT6A | 11 |
| GO:2000278 | BP | GO:2000278 | regulation of DNA biosynthetic process | 12/138 | 113/19623 | 2.43E-11 | 2.04E-09 | 1.58E-09 | CCT2/PTGES3/CCT5/HSP90AA1/CCT8/HSP90AB1/DSCC1/CCT4/CCT7/TCP1/DKC1/CCT6A | 12 |
| GO:0006403 | BP | GO:0006403 | RNA localization | 16/138 | 256/19623 | 3.57E-11 | 2.87E-09 | 2.22E-09 | CCT2/NUP107/CPSF6/XPOT/CCT5/RAN/CCT8/RAE1/CCT4/CCT7/TCP1/NPM1/SRSF1/DKC1/CCT6A/LRPPRC | 16 |
| GO:0032212 | BP | GO:0032212 | positive regulation of telomere maintenance via telomerase | 8/138 | 36/19623 | 1.25E-10 | 9.66E-09 | 7.47E-09 | CCT2/CCT5/CCT8/CCT4/CCT7/TCP1/DKC1/CCT6A | 8 |
| GO:1904358 | BP | GO:1904358 | positive regulation of telomere maintenance via telomere lengthening | 8/138 | 38/19623 | 2.00E-10 | 1.49E-08 | 1.15E-08 | CCT2/CCT5/CCT8/CCT4/CCT7/TCP1/DKC1/CCT6A | 8 |
| GO:0000723 | BP | GO:0000723 | telomere maintenance | 13/138 | 171/19623 | 2.42E-10 | 1.74E-08 | 1.35E-08 | CCT2/PTGES3/CCT5/HSP90AA1/CCT8/HSP90AB1/PRIM1/CCT4/CCT7/TCP1/DKC1/CCT6A/CCNE2 | 13 |
| GO:0034502 | BP | GO:0034502 | protein localization to chromosome | 10/138 | 82/19623 | 2.96E-10 | 2.05E-08 | 1.59E-08 | CCT2/CCT5/CCT8/CCT4/CDK1/CCT7/RAD21/TCP1/DKC1/CCT6A | 10 |
| GO:0032200 | BP | GO:0032200 | telomere organization | 13/138 | 184/19623 | 6.02E-10 | 4.03E-08 | 3.12E-08 | CCT2/PTGES3/CCT5/HSP90AA1/CCT8/HSP90AB1/PRIM1/CCT4/CCT7/TCP1/DKC1/CCT6A/CCNE2 | 13 |
| GO:1900182 | BP | GO:1900182 | positive regulation of protein localization to nucleus | 9/138 | 67/19623 | 9.79E-10 | 6.34E-08 | 4.91E-08 | CCT2/CCT5/CCT8/HSP90AB1/CCT4/CDK1/CCT7/TCP1/CCT6A | 9 |
| GO:0034504 | BP | GO:0034504 | protein localization to nucleus | 15/138 | 277/19623 | 1.11E-09 | 6.98E-08 | 5.41E-08 | CCT2/NUP107/CCT5/CSE1L/RAN/MDM2/CCT8/KPNA2/HSP90AB1/RAE1/CCT4/CDK1/CCT7/TCP1/CCT6A | 15 |
| GO:1902749 | BP | GO:1902749 | regulation of cell cycle G2/M phase transition | 14/138 | 250/19623 | 2.67E-09 | 1.63E-07 | 1.26E-07 | HSP90AA1/CCNB1/PSMD12/AURKA/CDK4/PSMD10/CDK1/PSMD14/RAD21/PSMA6/HMMR/NPM1/CDC25C/MAPRE1 | 14 |
| GO:0032210 | BP | GO:0032210 | regulation of telomere maintenance via telomerase | 8/138 | 56/19623 | 5.21E-09 | 3.08E-07 | 2.38E-07 | CCT2/CCT5/CCT8/CCT4/CCT7/TCP1/DKC1/CCT6A | 8 |
| GO:0032206 | BP | GO:0032206 | positive regulation of telomere maintenance | 8/138 | 60/19623 | 9.17E-09 | 5.26E-07 | 4.07E-07 | CCT2/CCT5/CCT8/CCT4/CCT7/TCP1/DKC1/CCT6A | 8 |
| GO:0051973 | BP | GO:0051973 | positive regulation of telomerase activity | 7/138 | 40/19623 | 1.12E-08 | 6.26E-07 | 4.84E-07 | CCT2/PTGES3/HSP90AA1/HSP90AB1/CCT4/TCP1/DKC1 | 7 |
| GO:0007059 | BP | GO:0007059 | chromosome segregation | 16/138 | 383/19623 | 1.25E-08 | 6.77E-07 | 5.24E-07 | NUP107/RAN/RACGAP1/MAD2L1/CCNB1/DSCC1/CDC6/RAD21/NCAPG/BIRC5/KIF18A/TOP1/LEMD3/SPAG5/MAPRE1/CCNE2 | 16 |
| GO:0098813 | BP | GO:0098813 | nuclear chromosome segregation | 15/138 | 333/19623 | 1.35E-08 | 7.03E-07 | 5.44E-07 | NUP107/RAN/RACGAP1/MAD2L1/CCNB1/DSCC1/CDC6/RAD21/NCAPG/BIRC5/KIF18A/LEMD3/SPAG5/MAPRE1/CCNE2 | 15 |
| GO:1904356 | BP | GO:1904356 | regulation of telomere maintenance via telomere lengthening | 8/138 | 63/19623 | 1.36E-08 | 7.03E-07 | 5.44E-07 | CCT2/CCT5/CCT8/CCT4/CCT7/TCP1/DKC1/CCT6A | 8 |
| GO:0033044 | BP | GO:0033044 | regulation of chromosome organization | 15/138 | 338/19623 | 1.65E-08 | 8.26E-07 | 6.40E-07 | CCT2/CCT5/UBE2N/MAD2L1/CCT8/CCNB1/SET/CDC6/CCT4/CCT7/RAD21/TCP1/DKC1/ATAD2/CCT6A | 15 |
| GO:0031647 | BP | GO:0031647 | regulation of protein stability | 14/138 | 290/19623 | 1.77E-08 | 8.69E-07 | 6.72E-07 | CCT2/PTGES3/HSPD1/CCT5/MDM2/HSP90AA1/CCT8/HSP90AB1/AURKA/CCT4/DERL1/CCT7/TCP1/CCT6A | 14 |
| GO:0000819 | BP | GO:0000819 | sister chromatid segregation | 13/138 | 249/19623 | 2.34E-08 | 1.12E-06 | 8.67E-07 | NUP107/RAN/RACGAP1/MAD2L1/CCNB1/DSCC1/CDC6/RAD21/NCAPG/BIRC5/KIF18A/SPAG5/MAPRE1 | 13 |
| GO:0044839 | BP | GO:0044839 | cell cycle G2/M phase transition | 14/138 | 302/19623 | 2.95E-08 | 1.38E-06 | 1.07E-06 | HSP90AA1/CCNB1/PSMD12/AURKA/CDK4/PSMD10/CDK1/PSMD14/RAD21/PSMA6/HMMR/NPM1/CDC25C/MAPRE1 | 14 |
| GO:1901990 | BP | GO:1901990 | regulation of mitotic cell cycle phase transition | 17/138 | 475/19623 | 4.12E-08 | 1.88E-06 | 1.45E-06 | MDM2/HSP90AA1/MAD2L1/CCNB1/PSMD12/AURKA/CDK4/PSMD10/CDC6/CDK1/PSMD14/RAD21/PSMA6/HMMR/NPM1/CDC25C/MAPRE1 | 17 |
| GO:0071897 | BP | GO:0071897 | DNA biosynthetic process | 12/138 | 218/19623 | 4.65E-08 | 2.08E-06 | 1.61E-06 | CCT2/PTGES3/CCT5/HSP90AA1/CCT8/HSP90AB1/DSCC1/CCT4/CCT7/TCP1/DKC1/CCT6A | 12 |
| GO:2001252 | BP | GO:2001252 | positive regulation of chromosome organization | 11/138 | 178/19623 | 5.33E-08 | 2.33E-06 | 1.80E-06 | CCT2/CCT5/UBE2N/CCT8/CCNB1/CCT4/CCT7/RAD21/TCP1/DKC1/CCT6A | 11 |
| GO:0051972 | BP | GO:0051972 | regulation of telomerase activity | 7/138 | 50/19623 | 5.67E-08 | 2.42E-06 | 1.87E-06 | CCT2/PTGES3/HSP90AA1/HSP90AB1/CCT4/TCP1/DKC1 | 7 |
| GO:0006913 | BP | GO:0006913 | nucleocytoplasmic transport | 15/138 | 384/19623 | 8.82E-08 | 3.62E-06 | 2.80E-06 | NUP107/CPSF6/XPOT/CSE1L/RAN/MDM2/HSPA9/KPNA2/HSP90AB1/RAE1/SET/CDK1/NPM1/SRSF1/PRKAG1 | 15 |
| GO:0050821 | BP | GO:0050821 | protein stabilization | 11/138 | 187/19623 | 8.82E-08 | 3.62E-06 | 2.80E-06 | CCT2/PTGES3/HSPD1/CCT5/HSP90AA1/CCT8/HSP90AB1/CCT4/CCT7/TCP1/CCT6A | 11 |
| GO:0010389 | BP | GO:0010389 | regulation of G2/M transition of mitotic cell cycle | 12/138 | 232/19623 | 9.21E-08 | 3.70E-06 | 2.86E-06 | HSP90AA1/CCNB1/PSMD12/AURKA/CDK4/PSMD10/CDK1/PSMD14/RAD21/PSMA6/HMMR/MAPRE1 | 12 |
| GO:0051169 | BP | GO:0051169 | nuclear transport | 15/138 | 388/19623 | 1.01E-07 | 3.97E-06 | 3.08E-06 | NUP107/CPSF6/XPOT/CSE1L/RAN/MDM2/HSPA9/KPNA2/HSP90AB1/RAE1/SET/CDK1/NPM1/SRSF1/PRKAG1 | 15 |
| GO:0000086 | BP | GO:0000086 | G2/M transition of mitotic cell cycle | 13/138 | 285/19623 | 1.14E-07 | 4.40E-06 | 3.40E-06 | HSP90AA1/CCNB1/PSMD12/AURKA/CDK4/PSMD10/CDK1/PSMD14/RAD21/PSMA6/HMMR/CDC25C/MAPRE1 | 13 |
| GO:1900180 | BP | GO:1900180 | regulation of protein localization to nucleus | 9/138 | 115/19623 | 1.21E-07 | 4.59E-06 | 3.55E-06 | CCT2/CCT5/CCT8/HSP90AB1/CCT4/CDK1/CCT7/TCP1/CCT6A | 9 |
| GO:0140014 | BP | GO:0140014 | mitotic nuclear division | 13/138 | 291/19623 | 1.45E-07 | 5.37E-06 | 4.16E-06 | RAN/RACGAP1/MAD2L1/CCNB1/DSCC1/AURKA/CDC6/RAD21/NCAPG/BIRC5/KIF18A/SPAG5/CDC25C | 13 |
| GO:0000070 | BP | GO:0000070 | mitotic sister chromatid segregation | 10/138 | 155/19623 | 1.47E-07 | 5.37E-06 | 4.16E-06 | RAN/RACGAP1/MAD2L1/CCNB1/DSCC1/CDC6/RAD21/NCAPG/KIF18A/SPAG5 | 10 |
| GO:0032204 | BP | GO:0032204 | regulation of telomere maintenance | 8/138 | 89/19623 | 2.13E-07 | 7.65E-06 | 5.92E-06 | CCT2/CCT5/CCT8/CCT4/CCT7/TCP1/DKC1/CCT6A | 8 |
| GO:1901998 | BP | GO:1901998 | toxin transport | 6/138 | 39/19623 | 2.92E-07 | 1.03E-05 | 7.97E-06 | CCT2/CCT5/CCT8/CCT4/CCT7/TCP1 | 6 |
| GO:1901991 | BP | GO:1901991 | negative regulation of mitotic cell cycle phase transition | 12/138 | 268/19623 | 4.36E-07 | 1.51E-05 | 1.17E-05 | MDM2/MAD2L1/CCNB1/PSMD12/AURKA/PSMD10/CDK1/PSMD14/RAD21/PSMA6/NPM1/CDC25C | 12 |
| GO:0031145 | BP | GO:0031145 | anaphase-promoting complex-dependent catabolic process | 8/138 | 99/19623 | 4.88E-07 | 1.66E-05 | 1.28E-05 | MAD2L1/CCNB1/PSMD12/AURKA/PSMD10/CDK1/PSMD14/PSMA6 | 8 |
| GO:0000280 | BP | GO:0000280 | nuclear division | 15/138 | 453/19623 | 7.28E-07 | 2.44E-05 | 1.89E-05 | RAN/RACGAP1/MAD2L1/CCNB1/DSCC1/AURKA/CDC6/RAD21/NCAPG/BIRC5/KIF18A/LEMD3/SPAG5/CDC25C/CCNE2 | 15 |
| GO:1901988 | BP | GO:1901988 | negative regulation of cell cycle phase transition | 12/138 | 288/19623 | 9.35E-07 | 3.08E-05 | 2.38E-05 | MDM2/MAD2L1/CCNB1/PSMD12/AURKA/PSMD10/CDK1/PSMD14/RAD21/PSMA6/NPM1/CDC25C | 12 |
| GO:0010948 | BP | GO:0010948 | negative regulation of cell cycle process | 14/138 | 404/19623 | 1.02E-06 | 3.31E-05 | 2.56E-05 | MDM2/MAD2L1/HSP90AB1/CCNB1/PSMD12/AURKA/CDK4/PSMD10/CDK1/PSMD14/RAD21/PSMA6/NPM1/CDC25C | 14 |
| GO:0007339 | BP | GO:0007339 | binding of sperm to zona pellucida | 6/138 | 48/19623 | 1.04E-06 | 3.33E-05 | 2.58E-05 | CCT2/CCT5/CCT8/CCT4/CCT7/TCP1 | 6 |
| GO:1902751 | BP | GO:1902751 | positive regulation of cell cycle G2/M phase transition | 5/138 | 27/19623 | 1.14E-06 | 3.58E-05 | 2.77E-05 | CCNB1/CDK4/CDK1/NPM1/CDC25C | 5 |
| GO:0000082 | BP | GO:0000082 | G1/S transition of mitotic cell cycle | 12/138 | 295/19623 | 1.20E-06 | 3.72E-05 | 2.88E-05 | MDM2/PPAT/CCNB1/PRIM1/AURKA/CDK4/CDC6/CDK1/NPM1/MCM4/CDC25C/CCNE2 | 12 |
| GO:0045787 | BP | GO:0045787 | positive regulation of cell cycle | 14/138 | 413/19623 | 1.32E-06 | 4.01E-05 | 3.10E-05 | MDM2/RACGAP1/MAD2L1/HSP90AB1/CCNB1/AURKA/CDK4/PSMD10/CDC6/CDK1/RAD21/NPM1/SPAG5/CDC25C | 14 |
| GO:0090068 | BP | GO:0090068 | positive regulation of cell cycle process | 12/138 | 298/19623 | 1.34E-06 | 4.01E-05 | 3.10E-05 | MDM2/RACGAP1/MAD2L1/CCNB1/AURKA/CDK4/CDC6/CDK1/RAD21/NPM1/SPAG5/CDC25C | 12 |
| GO:0051131 | BP | GO:0051131 | chaperone-mediated protein complex assembly | 5/138 | 28/19623 | 1.38E-06 | 4.08E-05 | 3.16E-05 | CCT2/PTGES3/HSPD1/HSP90AA1/HSP90AB1 | 5 |
| GO:0043487 | BP | GO:0043487 | regulation of RNA stability | 10/138 | 199/19623 | 1.47E-06 | 4.29E-05 | 3.32E-05 | PSMD12/PSMD10/YWHAZ/SET/PSMD14/PSMA6/NPM1/DKC1/PAIP1/YWHAB | 10 |
| GO:0060249 | BP | GO:0060249 | anatomical structure homeostasis | 14/138 | 426/19623 | 1.90E-06 | 5.47E-05 | 4.23E-05 | CCT2/PTGES3/CCT5/HSP90AA1/CCT8/HSP90AB1/PRIM1/CCT4/CCT7/STRAP/TCP1/DKC1/CCT6A/CCNE2 | 14 |
| GO:0006260 | BP | GO:0006260 | DNA replication | 12/138 | 310/19623 | 2.02E-06 | 5.71E-05 | 4.42E-05 | PRIM1/DSCC1/TIMELESS/SET/CDC6/CDK1/TOP1/MCM4/CACYBP/GINS1/CDC25C/CCNE2 | 12 |
| GO:0044843 | BP | GO:0044843 | cell cycle G1/S phase transition | 12/138 | 314/19623 | 2.30E-06 | 6.43E-05 | 4.97E-05 | MDM2/PPAT/CCNB1/PRIM1/AURKA/CDK4/CDC6/CDK1/NPM1/MCM4/CDC25C/CCNE2 | 12 |
| GO:0071156 | BP | GO:0071156 | regulation of cell cycle arrest | 8/138 | 123/19623 | 2.55E-06 | 7.01E-05 | 5.42E-05 | MDM2/HSP90AB1/CCNB1/AURKA/CDK4/CDK1/NPM1/CDC25C | 8 |
| GO:1903829 | BP | GO:1903829 | positive regulation of cellular protein localization | 13/138 | 378/19623 | 2.75E-06 | 7.48E-05 | 5.79E-05 | CCT2/CCT5/MDM2/CCT8/HSP90AB1/YWHAZ/CCT4/CDK1/CCT7/TCP1/DKC1/CCT6A/YWHAB | 13 |
| GO:0017038 | BP | GO:0017038 | protein import | 10/138 | 217/19623 | 3.21E-06 | 8.59E-05 | 6.65E-05 | NUP107/HSPD1/CSE1L/RAN/HSP90AA1/KPNA2/HSP90AB1/RAE1/CDK1/TIMM17A | 10 |
| GO:0051168 | BP | GO:0051168 | nuclear export | 10/138 | 223/19623 | 4.09E-06 | 0.00010805 | 8.36E-05 | NUP107/CPSF6/XPOT/CSE1L/RAN/MDM2/HSPA9/RAE1/NPM1/SRSF1 | 10 |
| GO:1901989 | BP | GO:1901989 | positive regulation of cell cycle phase transition | 7/138 | 93/19623 | 4.18E-06 | 0.00010907 | 8.44E-05 | MDM2/CCNB1/CDK4/CDC6/CDK1/NPM1/CDC25C | 7 |
| GO:1900034 | BP | GO:1900034 | regulation of cellular response to heat | 7/138 | 96/19623 | 5.17E-06 | 0.000133116 | 0.000103017 | NUP107/PTGES3/HSP90AA1/HSP90AB1/RAE1/FKBP4/HSPH1 | 7 |
| GO:0035036 | BP | GO:0035036 | sperm-egg recognition | 6/138 | 64/19623 | 5.82E-06 | 0.000147932 | 0.000114483 | CCT2/CCT5/CCT8/CCT4/CCT7/TCP1 | 6 |
| GO:0043488 | BP | GO:0043488 | regulation of mRNA stability | 9/138 | 185/19623 | 6.53E-06 | 0.000164015 | 0.000126929 | PSMD12/PSMD10/YWHAZ/SET/PSMD14/PSMA6/NPM1/PAIP1/YWHAB | 9 |
| GO:0097064 | BP | GO:0097064 | ncRNA export from nucleus | 5/138 | 39/19623 | 7.60E-06 | 0.000188458 | 0.000145845 | NUP107/XPOT/RAN/RAE1/NPM1 | 5 |
| GO:0006977 | BP | GO:0006977 | DNA damage response, signal transduction by p53 class mediator resulting in cell cycle arrest | 6/138 | 71/19623 | 1.07E-05 | 0.000261497 | 0.000202369 | MDM2/CCNB1/AURKA/CDK1/NPM1/CDC25C | 6 |
| GO:0072431 | BP | GO:0072431 | signal transduction involved in mitotic G1 DNA damage checkpoint | 6/138 | 72/19623 | 1.16E-05 | 0.00027688 | 0.000214273 | MDM2/CCNB1/AURKA/CDK1/NPM1/CDC25C | 6 |
| GO:1902400 | BP | GO:1902400 | intracellular signal transduction involved in G1 DNA damage checkpoint | 6/138 | 72/19623 | 1.16E-05 | 0.00027688 | 0.000214273 | MDM2/CCNB1/AURKA/CDK1/NPM1/CDC25C | 6 |
| GO:0006611 | BP | GO:0006611 | protein export from nucleus | 9/138 | 201/19623 | 1.27E-05 | 0.000301026 | 0.00023296 | NUP107/XPOT/CSE1L/RAN/MDM2/HSPA9/RAE1/NPM1/SRSF1 | 9 |
| GO:0072413 | BP | GO:0072413 | signal transduction involved in mitotic cell cycle checkpoint | 6/138 | 74/19623 | 1.36E-05 | 0.000309786 | 0.000239739 | MDM2/CCNB1/AURKA/CDK1/NPM1/CDC25C | 6 |
| GO:1902402 | BP | GO:1902402 | signal transduction involved in mitotic DNA damage checkpoint | 6/138 | 74/19623 | 1.36E-05 | 0.000309786 | 0.000239739 | MDM2/CCNB1/AURKA/CDK1/NPM1/CDC25C | 6 |
| GO:1902403 | BP | GO:1902403 | signal transduction involved in mitotic DNA integrity checkpoint | 6/138 | 74/19623 | 1.36E-05 | 0.000309786 | 0.000239739 | MDM2/CCNB1/AURKA/CDK1/NPM1/CDC25C | 6 |
| GO:0045930 | BP | GO:0045930 | negative regulation of mitotic cell cycle | 12/138 | 376/19623 | 1.43E-05 | 0.000323544 | 0.000250386 | MDM2/MAD2L1/CCNB1/PSMD12/AURKA/PSMD10/CDK1/PSMD14/RAD21/PSMA6/NPM1/CDC25C | 12 |
| GO:0034605 | BP | GO:0034605 | cellular response to heat | 8/138 | 156/19623 | 1.48E-05 | 0.000329523 | 0.000255013 | NUP107/PTGES3/HSP90AA1/HSPA9/HSP90AB1/RAE1/FKBP4/HSPH1 | 8 |
| GO:0061013 | BP | GO:0061013 | regulation of mRNA catabolic process | 9/138 | 206/19623 | 1.55E-05 | 0.000342077 | 0.000264729 | PSMD12/PSMD10/YWHAZ/SET/PSMD14/PSMA6/NPM1/PAIP1/YWHAB | 9 |
| GO:0009408 | BP | GO:0009408 | response to heat | 9/138 | 211/19623 | 1.87E-05 | 0.000409379 | 0.000316813 | NUP107/PTGES3/HSP90AA1/HSPA9/HSP90AB1/RAE1/FKBP4/HSPH1/DNAJA1 | 9 |
| GO:0007050 | BP | GO:0007050 | cell cycle arrest | 10/138 | 266/19623 | 1.91E-05 | 0.000412467 | 0.000319203 | MDM2/PA2G4/HSP90AB1/CCNB1/AURKA/CDK4/CDK1/NPM1/CDC25C/PRKAG1 | 10 |
| GO:0043161 | BP | GO:0043161 | proteasome-mediated ubiquitin-dependent protein catabolic process | 13/138 | 455/19623 | 2.01E-05 | 0.000425261 | 0.000329104 | MDM2/MAD2L1/HSP90AB1/CCNB1/PSMD12/UBE2V2/AURKA/PSMD10/FBXO45/CDK1/PSMD14/DERL1/PSMA6 | 13 |
| GO:0010972 | BP | GO:0010972 | negative regulation of G2/M transition of mitotic cell cycle | 7/138 | 118/19623 | 2.01E-05 | 0.000425261 | 0.000329104 | PSMD12/AURKA/PSMD10/CDK1/PSMD14/RAD21/PSMA6 | 7 |
| GO:0031571 | BP | GO:0031571 | mitotic G1 DNA damage checkpoint | 6/138 | 80/19623 | 2.13E-05 | 0.000440326 | 0.000340762 | MDM2/CCNB1/AURKA/CDK1/NPM1/CDC25C | 6 |
| GO:0044819 | BP | GO:0044819 | mitotic G1/S transition checkpoint | 6/138 | 80/19623 | 2.13E-05 | 0.000440326 | 0.000340762 | MDM2/CCNB1/AURKA/CDK1/NPM1/CDC25C | 6 |
| GO:0051084 | BP | GO:0051084 | de novo' posttranslational protein folding | 5/138 | 48/19623 | 2.15E-05 | 0.000440326 | 0.000340762 | CCT2/PTGES3/HSPA9/HSPH1/HSPE1 | 5 |
| GO:0044783 | BP | GO:0044783 | G1 DNA damage checkpoint | 6/138 | 81/19623 | 2.28E-05 | 0.000463378 | 0.000358602 | MDM2/CCNB1/AURKA/CDK1/NPM1/CDC25C | 6 |
| GO:0030330 | BP | GO:0030330 | DNA damage response, signal transduction by p53 class mediator | 7/138 | 121/19623 | 2.37E-05 | 0.000475448 | 0.000367942 | MDM2/CCNB1/AURKA/PSMD10/CDK1/NPM1/CDC25C | 7 |
| GO:0009266 | BP | GO:0009266 | response to temperature stimulus | 10/138 | 275/19623 | 2.54E-05 | 0.000505041 | 0.000390844 | NUP107/PTGES3/HSPD1/HSP90AA1/HSPA9/HSP90AB1/RAE1/FKBP4/HSPH1/DNAJA1 | 10 |
| GO:0009988 | BP | GO:0009988 | cell-cell recognition | 6/138 | 83/19623 | 2.63E-05 | 0.000517089 | 0.000400168 | CCT2/CCT5/CCT8/CCT4/CCT7/TCP1 | 6 |
| GO:0070585 | BP | GO:0070585 | protein localization to mitochondrion | 9/138 | 223/19623 | 2.90E-05 | 0.000565677 | 0.000437769 | HSPD1/HSP90AA1/YWHAZ/HSPH1/TOMM70/TIMM17A/MTCH2/DNAJA1/YWHAB | 9 |
| GO:0072401 | BP | GO:0072401 | signal transduction involved in DNA integrity checkpoint | 6/138 | 87/19623 | 3.43E-05 | 0.000656768 | 0.000508264 | MDM2/CCNB1/AURKA/CDK1/NPM1/CDC25C | 6 |
| GO:0072422 | BP | GO:0072422 | signal transduction involved in DNA damage checkpoint | 6/138 | 87/19623 | 3.43E-05 | 0.000656768 | 0.000508264 | MDM2/CCNB1/AURKA/CDK1/NPM1/CDC25C | 6 |
| GO:0072395 | BP | GO:0072395 | signal transduction involved in cell cycle checkpoint | 6/138 | 88/19623 | 3.66E-05 | 0.000694176 | 0.000537213 | MDM2/CCNB1/AURKA/CDK1/NPM1/CDC25C | 6 |
| GO:1902750 | BP | GO:1902750 | negative regulation of cell cycle G2/M phase transition | 7/138 | 130/19623 | 3.76E-05 | 0.00070546 | 0.000545946 | PSMD12/AURKA/PSMD10/CDK1/PSMD14/RAD21/PSMA6 | 7 |
| GO:0000079 | BP | GO:0000079 | regulation of cyclin-dependent protein serine/threonine kinase activity | 6/138 | 89/19623 | 3.90E-05 | 0.000726387 | 0.000562141 | CCNB1/CDK4/PSMD10/CDC6/CDC25C/CCNE2 | 6 |
| GO:0051170 | BP | GO:0051170 | import into nucleus | 8/138 | 181/19623 | 4.29E-05 | 0.000791489 | 0.000612522 | NUP107/CSE1L/RAN/KPNA2/HSP90AB1/RAE1/CDK1/PRKAG1 | 8 |
| GO:1904029 | BP | GO:1904029 | regulation of cyclin-dependent protein kinase activity | 6/138 | 95/19623 | 5.64E-05 | 0.001030509 | 0.000797496 | CCNB1/CDK4/PSMD10/CDC6/CDC25C/CCNE2 | 6 |
| GO:0007062 | BP | GO:0007062 | sister chromatid cohesion | 7/138 | 141/19623 | 6.30E-05 | 0.001137625 | 0.000880392 | NUP107/MAD2L1/DSCC1/RAD21/BIRC5/KIF18A/MAPRE1 | 7 |
| GO:0071158 | BP | GO:0071158 | positive regulation of cell cycle arrest | 6/138 | 97/19623 | 6.34E-05 | 0.001137625 | 0.000880392 | MDM2/CCNB1/AURKA/CDK1/NPM1/CDC25C | 6 |
| GO:2000045 | BP | GO:2000045 | regulation of G1/S transition of mitotic cell cycle | 8/138 | 193/19623 | 6.75E-05 | 0.001200943 | 0.000929393 | MDM2/CCNB1/AURKA/CDK4/CDC6/CDK1/NPM1/CDC25C | 8 |
| GO:0042770 | BP | GO:0042770 | signal transduction in response to DNA damage | 7/138 | 145/19623 | 7.52E-05 | 0.001325923 | 0.001026113 | MDM2/CCNB1/AURKA/PSMD10/CDK1/NPM1/CDC25C | 7 |
| GO:0051983 | BP | GO:0051983 | regulation of chromosome segregation | 6/138 | 101/19623 | 7.95E-05 | 0.001388702 | 0.001074697 | RACGAP1/MAD2L1/CCNB1/CDC6/RAD21/SPAG5 | 6 |
| GO:0042254 | BP | GO:0042254 | ribosome biogenesis | 11/138 | 381/19623 | 8.14E-05 | 0.001410601 | 0.001091644 | RAN/DCAF13/PA2G4/NOL11/UTP23/PNO1/BRIX1/NPM1/DKC1/UTP18/NOLC1 | 11 |
| GO:0031570 | BP | GO:0031570 | DNA integrity checkpoint | 8/138 | 200/19623 | 8.67E-05 | 0.001488064 | 0.001151592 | MDM2/CCNB1/AURKA/TIMELESS/CDC6/CDK1/NPM1/CDC25C | 8 |
| GO:0006409 | BP | GO:0006409 | tRNA export from nucleus | 4/138 | 34/19623 | 9.22E-05 | 0.001542109 | 0.001193416 | NUP107/XPOT/RAN/RAE1 | 4 |
| GO:0071431 | BP | GO:0071431 | tRNA-containing ribonucleoprotein complex export from nucleus | 4/138 | 34/19623 | 9.22E-05 | 0.001542109 | 0.001193416 | NUP107/XPOT/RAN/RAE1 | 4 |
| GO:0000075 | BP | GO:0000075 | cell cycle checkpoint | 9/138 | 259/19623 | 9.23E-05 | 0.001542109 | 0.001193416 | MDM2/MAD2L1/CCNB1/AURKA/TIMELESS/CDC6/CDK1/NPM1/CDC25C | 9 |
| GO:0006986 | BP | GO:0006986 | response to unfolded protein | 8/138 | 202/19623 | 9.29E-05 | 0.001542109 | 0.001193416 | HSPD1/HSP90AA1/HSPA9/HSP90AB1/HSPH1/DERL1/DNAJA1/HSPE1 | 8 |
| GO:1903311 | BP | GO:1903311 | regulation of mRNA metabolic process | 10/138 | 325/19623 | 0.000102931 | 0.001694995 | 0.001311733 | CCNB1/PSMD12/PSMD10/YWHAZ/SET/PSMD14/PSMA6/NPM1/PAIP1/YWHAB | 10 |
| GO:0006261 | BP | GO:0006261 | DNA-dependent DNA replication | 7/138 | 154/19623 | 0.000109793 | 0.00179329 | 0.001387802 | PRIM1/DSCC1/TIMELESS/CDC6/MCM4/GINS1/CCNE2 | 7 |
| GO:0051031 | BP | GO:0051031 | tRNA transport | 4/138 | 36/19623 | 0.000115818 | 0.001876434 | 0.001452146 | NUP107/XPOT/RAN/RAE1 | 4 |
| GO:0051988 | BP | GO:0051988 | regulation of attachment of spindle microtubules to kinetochore | 3/138 | 14/19623 | 0.000117039 | 0.001881053 | 0.00145572 | RACGAP1/CCNB1/SPAG5 | 3 |
| GO:0006606 | BP | GO:0006606 | protein import into nucleus | 7/138 | 157/19623 | 0.000123829 | 0.001974379 | 0.001527944 | NUP107/CSE1L/RAN/KPNA2/HSP90AB1/RAE1/CDK1 | 7 |
| GO:1902806 | BP | GO:1902806 | regulation of cell cycle G1/S phase transition | 8/138 | 211/19623 | 0.000125625 | 0.001987253 | 0.001537907 | MDM2/CCNB1/AURKA/CDK4/CDC6/CDK1/NPM1/CDC25C | 8 |
| GO:0006405 | BP | GO:0006405 | RNA export from nucleus | 7/138 | 158/19623 | 0.000128818 | 0.002021845 | 0.001564677 | NUP107/CPSF6/XPOT/RAN/RAE1/NPM1/SRSF1 | 7 |
| GO:0007338 | BP | GO:0007338 | single fertilization | 7/138 | 161/19623 | 0.000144776 | 0.002254686 | 0.00174487 | CCT2/CCT5/CCT8/CCT4/CDK1/CCT7/TCP1 | 7 |
| GO:0050657 | BP | GO:0050657 | nucleic acid transport | 8/138 | 217/19623 | 0.000152353 | 0.002336461 | 0.001808154 | NUP107/CPSF6/XPOT/RAN/RAE1/NPM1/SRSF1/LRPPRC | 8 |
| GO:0050658 | BP | GO:0050658 | RNA transport | 8/138 | 217/19623 | 0.000152353 | 0.002336461 | 0.001808154 | NUP107/CPSF6/XPOT/RAN/RAE1/NPM1/SRSF1/LRPPRC | 8 |
| GO:0045931 | BP | GO:0045931 | positive regulation of mitotic cell cycle | 7/138 | 163/19623 | 0.000156278 | 0.002378499 | 0.001840687 | MDM2/MAD2L1/CCNB1/AURKA/CDK4/CDC6/CDK1 | 7 |
| GO:0072655 | BP | GO:0072655 | establishment of protein localization to mitochondrion | 8/138 | 219/19623 | 0.00016224 | 0.002450675 | 0.001896543 | HSPD1/HSP90AA1/YWHAZ/HSPH1/TOMM70/TIMM17A/DNAJA1/YWHAB | 8 |
| GO:0051236 | BP | GO:0051236 | establishment of RNA localization | 8/138 | 220/19623 | 0.000167378 | 0.002509423 | 0.001942007 | NUP107/CPSF6/XPOT/RAN/RAE1/NPM1/SRSF1/LRPPRC | 8 |
| GO:0071456 | BP | GO:0071456 | cellular response to hypoxia | 8/138 | 223/19623 | 0.000183604 | 0.002732294 | 0.002114484 | MDM2/CCNB1/PSMD12/ELOC/PSMD10/PSMD14/PSMA6/PGK1 | 8 |
| GO:0042026 | BP | GO:0042026 | protein refolding | 4/138 | 41/19623 | 0.000193769 | 0.002862365 | 0.002215144 | HSPD1/HSP90AA1/HSPA9/HSPH1 | 4 |
| GO:0051085 | BP | GO:0051085 | chaperone cofactor-dependent protein refolding | 4/138 | 42/19623 | 0.000213004 | 0.003123535 | 0.00241726 | PTGES3/HSPA9/HSPH1/HSPE1 | 4 |
| GO:0009127 | BP | GO:0009127 | purine nucleoside monophosphate biosynthetic process | 7/138 | 172/19623 | 0.000217567 | 0.00314455 | 0.002433523 | NUP107/ATP5F1B/PAICS/PPAT/RAE1/PGK1/PRKAG1 | 7 |
| GO:0009168 | BP | GO:0009168 | purine ribonucleoside monophosphate biosynthetic process | 7/138 | 172/19623 | 0.000217567 | 0.00314455 | 0.002433523 | NUP107/ATP5F1B/PAICS/PPAT/RAE1/PGK1/PRKAG1 | 7 |
| GO:0036294 | BP | GO:0036294 | cellular response to decreased oxygen levels | 8/138 | 230/19623 | 0.000226543 | 0.003250887 | 0.002515816 | MDM2/CCNB1/PSMD12/ELOC/PSMD10/PSMD14/PSMA6/PGK1 | 8 |
| GO:0006270 | BP | GO:0006270 | DNA replication initiation | 4/138 | 43/19623 | 0.000233576 | 0.003328048 | 0.00257553 | PRIM1/CDC6/MCM4/CCNE2 | 4 |
| GO:0042274 | BP | GO:0042274 | ribosomal small subunit biogenesis | 5/138 | 79/19623 | 0.000237479 | 0.003336325 | 0.002581935 | DCAF13/NOL11/UTP23/NPM1/UTP18 | 5 |
| GO:1901992 | BP | GO:1901992 | positive regulation of mitotic cell cycle phase transition | 5/138 | 79/19623 | 0.000237479 | 0.003336325 | 0.002581935 | MDM2/CCNB1/CDK4/CDC6/CDK1 | 5 |
| GO:0035966 | BP | GO:0035966 | response to topologically incorrect protein | 8/138 | 233/19623 | 0.000247308 | 0.003450294 | 0.002670134 | HSPD1/HSP90AA1/HSPA9/HSP90AB1/HSPH1/DERL1/DNAJA1/HSPE1 | 8 |
| GO:0006521 | BP | GO:0006521 | regulation of cellular amino acid metabolic process | 5/138 | 80/19623 | 0.000251893 | 0.003490018 | 0.002700876 | PSMD12/AZIN1/PSMD10/PSMD14/PSMA6 | 5 |
| GO:0007077 | BP | GO:0007077 | mitotic nuclear envelope disassembly | 4/138 | 45/19623 | 0.000278951 | 0.00381785 | 0.00295458 | NUP107/CCNB1/RAE1/CDK1 | 4 |
| GO:2000134 | BP | GO:2000134 | negative regulation of G1/S transition of mitotic cell cycle | 6/138 | 127/19623 | 0.000279355 | 0.00381785 | 0.00295458 | MDM2/CCNB1/AURKA/CDK1/NPM1/CDC25C | 6 |
| GO:0022618 | BP | GO:0022618 | ribonucleoprotein complex assembly | 8/138 | 238/19623 | 0.000285371 | 0.003873719 | 0.002997817 | PTGES3/HSP90AA1/DENR/HSP90AB1/BRIX1/STRAP/NPM1/SRSF1 | 8 |
| GO:0031100 | BP | GO:0031100 | animal organ regeneration | 5/138 | 84/19623 | 0.0003163 | 0.004242721 | 0.003283382 | PPAT/AURKA/CDK4/CDK1/SRSF1 | 5 |
| GO:0044773 | BP | GO:0044773 | mitotic DNA damage checkpoint | 6/138 | 130/19623 | 0.000316779 | 0.004242721 | 0.003283382 | MDM2/CCNB1/AURKA/CDK1/NPM1/CDC25C | 6 |
| GO:0009156 | BP | GO:0009156 | ribonucleoside monophosphate biosynthetic process | 7/138 | 185/19623 | 0.000338968 | 0.004509846 | 0.003490106 | NUP107/ATP5F1B/PAICS/PPAT/RAE1/PGK1/PRKAG1 | 7 |
| GO:0071453 | BP | GO:0071453 | cellular response to oxygen levels | 8/138 | 246/19623 | 0.000356148 | 0.004675549 | 0.003618341 | MDM2/CCNB1/PSMD12/ELOC/PSMD10/PSMD14/PSMA6/PGK1 | 8 |
| GO:0007052 | BP | GO:0007052 | mitotic spindle organization | 6/138 | 133/19623 | 0.000358025 | 0.004675549 | 0.003618341 | RAN/RACGAP1/CCNB1/RAE1/AURKA/BIRC5 | 6 |
| GO:0030071 | BP | GO:0030071 | regulation of mitotic metaphase/anaphase transition | 4/138 | 48/19623 | 0.000358404 | 0.004675549 | 0.003618341 | MAD2L1/CCNB1/CDC6/RAD21 | 4 |
| GO:0006998 | BP | GO:0006998 | nuclear envelope organization | 5/138 | 87/19623 | 0.000372256 | 0.004799318 | 0.003714125 | NUP107/CCNB1/RAE1/CDK1/LEMD3 | 5 |
| GO:1902807 | BP | GO:1902807 | negative regulation of cell cycle G1/S phase transition | 6/138 | 134/19623 | 0.00037267 | 0.004799318 | 0.003714125 | MDM2/CCNB1/AURKA/CDK1/NPM1/CDC25C | 6 |
| GO:0030397 | BP | GO:0030397 | membrane disassembly | 4/138 | 49/19623 | 0.000388148 | 0.004904339 | 0.003795399 | NUP107/CCNB1/RAE1/CDK1 | 4 |
| GO:0051081 | BP | GO:0051081 | nuclear envelope disassembly | 4/138 | 49/19623 | 0.000388148 | 0.004904339 | 0.003795399 | NUP107/CCNB1/RAE1/CDK1 | 4 |
| GO:1902099 | BP | GO:1902099 | regulation of metaphase/anaphase transition of cell cycle | 4/138 | 49/19623 | 0.000388148 | 0.004904339 | 0.003795399 | MAD2L1/CCNB1/CDC6/RAD21 | 4 |
| GO:0071826 | BP | GO:0071826 | ribonucleoprotein complex subunit organization | 8/138 | 250/19623 | 0.000396561 | 0.004979321 | 0.003853427 | PTGES3/HSP90AA1/DENR/HSP90AB1/BRIX1/STRAP/NPM1/SRSF1 | 8 |
| GO:0070507 | BP | GO:0070507 | regulation of microtubule cytoskeleton organization | 7/138 | 191/19623 | 0.000410835 | 0.005126503 | 0.003967328 | RAE1/AURKA/FKBP4/NPM1/KIF18A/SPAG5/MAPRE1 | 7 |
| GO:0006401 | BP | GO:0006401 | RNA catabolic process | 11/138 | 460/19623 | 0.000415033 | 0.005146924 | 0.003983132 | PSMD12/PSMD10/YWHAZ/SET/PSMD14/PSMA6/NPM1/DKC1/LRPPRC/PAIP1/YWHAB | 11 |
| GO:0044774 | BP | GO:0044774 | mitotic DNA integrity checkpoint | 6/138 | 137/19623 | 0.000419432 | 0.005169562 | 0.004000651 | MDM2/CCNB1/AURKA/CDK1/NPM1/CDC25C | 6 |
| GO:0007093 | BP | GO:0007093 | mitotic cell cycle checkpoint | 7/138 | 193/19623 | 0.000437327 | 0.005357256 | 0.004145905 | MDM2/MAD2L1/CCNB1/AURKA/CDK1/NPM1/CDC25C | 7 |
| GO:0007091 | BP | GO:0007091 | metaphase/anaphase transition of mitotic cell cycle | 4/138 | 51/19623 | 0.000452861 | 0.005511251 | 0.004265079 | MAD2L1/CCNB1/CDC6/RAD21 | 4 |
| GO:0044766 | BP | GO:0044766 | multi-organism transport | 5/138 | 91/19623 | 0.000458128 | 0.005511251 | 0.004265079 | NUP107/RAN/KPNA2/RAE1/TCP1 | 5 |
| GO:1902579 | BP | GO:1902579 | multi-organism localization | 5/138 | 91/19623 | 0.000458128 | 0.005511251 | 0.004265079 | NUP107/RAN/KPNA2/RAE1/TCP1 | 5 |
| GO:0009124 | BP | GO:0009124 | nucleoside monophosphate biosynthetic process | 7/138 | 195/19623 | 0.000465167 | 0.005562627 | 0.004304839 | NUP107/ATP5F1B/PAICS/PPAT/RAE1/PGK1/PRKAG1 | 7 |
| GO:1903578 | BP | GO:1903578 | regulation of ATP metabolic process | 5/138 | 92/19623 | 0.00048175 | 0.005726842 | 0.004431922 | NUP107/CCNB1/RAE1/CDK1/PRKAG1 | 5 |
| GO:0044784 | BP | GO:0044784 | metaphase/anaphase transition of cell cycle | 4/138 | 52/19623 | 0.000487943 | 0.005742354 | 0.004443927 | MAD2L1/CCNB1/CDC6/RAD21 | 4 |
| GO:0071426 | BP | GO:0071426 | ribonucleoprotein complex export from nucleus | 6/138 | 141/19623 | 0.000488772 | 0.005742354 | 0.004443927 | NUP107/XPOT/RAN/RAE1/NPM1/SRSF1 | 6 |
| GO:0031503 | BP | GO:0031503 | protein-containing complex localization | 8/138 | 260/19623 | 0.000514134 | 0.006005207 | 0.004647345 | NUP107/XPOT/RAN/RAE1/FKBP4/NPM1/BIRC5/SRSF1 | 8 |
| GO:0009566 | BP | GO:0009566 | fertilization | 7/138 | 199/19623 | 0.000525091 | 0.006088174 | 0.004711552 | CCT2/CCT5/CCT8/CCT4/CDK1/CCT7/TCP1 | 7 |
| GO:0015931 | BP | GO:0015931 | nucleobase-containing compound transport | 8/138 | 261/19623 | 0.000527298 | 0.006088174 | 0.004711552 | NUP107/CPSF6/XPOT/RAN/RAE1/NPM1/SRSF1/LRPPRC | 8 |
| GO:0010971 | BP | GO:0010971 | positive regulation of G2/M transition of mitotic cell cycle | 3/138 | 23/19623 | 0.00054365 | 0.006241097 | 0.004829898 | CCNB1/CDK4/CDK1 | 3 |
| GO:0031396 | BP | GO:0031396 | regulation of protein ubiquitination | 7/138 | 201/19623 | 0.000557275 | 0.006361166 | 0.004922817 | UBE2N/HSP90AA1/MAD2L1/HSP90AB1/PSMD10/DERL1/DNAJA1 | 7 |
| GO:0010965 | BP | GO:0010965 | regulation of mitotic sister chromatid separation | 4/138 | 54/19623 | 0.000563844 | 0.006399788 | 0.004952706 | MAD2L1/CCNB1/CDC6/RAD21 | 4 |
| GO:0016925 | BP | GO:0016925 | protein sumoylation | 5/138 | 96/19623 | 0.000585519 | 0.006608472 | 0.005114204 | NUP107/MDM2/RAE1/BIRC5/TOP1 | 5 |
| GO:0071157 | BP | GO:0071157 | negative regulation of cell cycle arrest | 3/138 | 24/19623 | 0.000618127 | 0.00693753 | 0.005368857 | MDM2/HSP90AB1/CDK4 | 3 |
| GO:0051306 | BP | GO:0051306 | mitotic sister chromatid separation | 4/138 | 56/19623 | 0.000647775 | 0.007229884 | 0.005595105 | MAD2L1/CCNB1/CDC6/RAD21 | 4 |
| GO:0016072 | BP | GO:0016072 | rRNA metabolic process | 9/138 | 339/19623 | 0.000671176 | 0.007436871 | 0.00575529 | DCAF13/PA2G4/NOL11/UTP23/PNO1/DKC1/UTP18/MARS/NOLC1 | 9 |
| GO:0061418 | BP | GO:0061418 | regulation of transcription from RNA polymerase II promoter in response to hypoxia | 5/138 | 99/19623 | 0.000673724 | 0.007436871 | 0.00575529 | PSMD12/ELOC/PSMD10/PSMD14/PSMA6 | 5 |
| GO:0006997 | BP | GO:0006997 | nucleus organization | 6/138 | 151/19623 | 0.000701422 | 0.007658359 | 0.005926697 | NUP107/CCNB1/RAE1/CDK1/LEMD3/NOLC1 | 6 |
| GO:0071166 | BP | GO:0071166 | ribonucleoprotein complex localization | 6/138 | 151/19623 | 0.000701422 | 0.007658359 | 0.005926697 | NUP107/XPOT/RAN/RAE1/NPM1/SRSF1 | 6 |
| GO:0033238 | BP | GO:0033238 | regulation of cellular amine metabolic process | 5/138 | 100/19623 | 0.000705225 | 0.007658359 | 0.005926697 | PSMD12/AZIN1/PSMD10/PSMD14/PSMA6 | 5 |
| GO:0046824 | BP | GO:0046824 | positive regulation of nucleocytoplasmic transport | 4/138 | 58/19623 | 0.000740196 | 0.007934977 | 0.006140767 | CPSF6/MDM2/HSP90AB1/CDK1 | 4 |
| GO:1905818 | BP | GO:1905818 | regulation of chromosome separation | 4/138 | 58/19623 | 0.000740196 | 0.007934977 | 0.006140767 | MAD2L1/CCNB1/CDC6/RAD21 | 4 |
| GO:0038093 | BP | GO:0038093 | Fc receptor signaling pathway | 7/138 | 211/19623 | 0.000742546 | 0.007934977 | 0.006140767 | UBE2N/HSP90AA1/HSP90AB1/PSMD12/PSMD10/PSMD14/PSMA6 | 7 |
| GO:1902850 | BP | GO:1902850 | microtubule cytoskeleton organization involved in mitosis | 6/138 | 154/19623 | 0.000777435 | 0.008263845 | 0.006395274 | RAN/RACGAP1/CCNB1/RAE1/AURKA/BIRC5 | 6 |
| GO:0030490 | BP | GO:0030490 | maturation of SSU-rRNA | 4/138 | 59/19623 | 0.000789736 | 0.008350415 | 0.006462269 | DCAF13/NOL11/UTP23/UTP18 | 4 |
| GO:0006520 | BP | GO:0006520 | cellular amino acid metabolic process | 10/138 | 422/19623 | 0.000815581 | 0.008578548 | 0.006638817 | PPAT/PSMD12/AZIN1/ENOPH1/PSMD10/PSMD14/PSMA6/MARS/RARS/TARS | 10 |
| GO:0032886 | BP | GO:0032886 | regulation of microtubule-based process | 7/138 | 220/19623 | 0.000948185 | 0.009921377 | 0.007678014 | RAE1/AURKA/FKBP4/NPM1/KIF18A/SPAG5/MAPRE1 | 7 |
| GO:0005832 | CC | GO:0005832 | chaperonin-containing T-complex | 7/139 | 12/20758 | 3.99E-13 | 1.25E-10 | 9.29E-11 | CCT2/CCT5/CCT8/CCT4/CCT7/TCP1/CCT6A | 7 |
| GO:0101031 | CC | GO:0101031 | chaperone complex | 7/139 | 13/20758 | 8.60E-13 | 1.34E-10 | 1.00E-10 | CCT2/CCT5/CCT8/CCT4/CCT7/TCP1/CCT6A | 7 |
| GO:0002199 | CC | GO:0002199 | zona pellucida receptor complex | 6/139 | 13/20758 | 1.34E-10 | 1.08E-08 | 8.02E-09 | CCT2/CCT5/CCT8/CCT4/CCT7/TCP1 | 6 |
| GO:0005874 | CC | GO:0005874 | microtubule | 19/139 | 443/20758 | 1.38E-10 | 1.08E-08 | 8.02E-09 | CCT2/CCT5/RACGAP1/CCT8/TUBA1B/AURKA/FKBP4/TUBA1C/HSPH1/CCT4/CDK1/CCT7/TCP1/BIRC5/KIF18A/SPAG5/CCT6A/LRPPRC/MAPRE1 | 19 |
| GO:0043209 | CC | GO:0043209 | myelin sheath | 10/139 | 177/20758 | 3.25E-07 | 2.03E-05 | 1.51E-05 | CCT2/HSPD1/CCT5/ATP5F1B/HSP90AA1/HSPA9/TUBA1B/STIP1/VDAC1/TCP1 | 10 |
| GO:0000775 | CC | GO:0000775 | chromosome, centromeric region | 10/139 | 208/20758 | 1.42E-06 | 7.41E-05 | 5.52E-05 | NUP107/MAD2L1/CCNB1/DSCC1/AURKA/RAD21/NCAPG/BIRC5/KIF18A/SPAG5 | 10 |
| GO:0098687 | CC | GO:0098687 | chromosomal region | 13/139 | 383/20758 | 1.87E-06 | 8.36E-05 | 6.23E-05 | NUP107/PTGES3/MAD2L1/CCNB1/DSCC1/AURKA/CDK1/RAD21/NCAPG/BIRC5/KIF18A/MCM4/SPAG5 | 13 |
| GO:0005819 | CC | GO:0005819 | spindle | 12/139 | 343/20758 | 3.49E-06 | 0.000135926 | 0.000101349 | RACGAP1/MAD2L1/CCNB1/RAE1/AURKA/CDC6/CDK1/NPM1/BIRC5/KIF18A/SPAG5/MAPRE1 | 12 |
| GO:0072686 | CC | GO:0072686 | mitotic spindle | 7/139 | 104/20758 | 6.42E-06 | 0.000222406 | 0.000165829 | RACGAP1/MAD2L1/RAE1/AURKA/CDK1/KIF18A/SPAG5 | 7 |
| GO:0000307 | CC | GO:0000307 | cyclin-dependent protein kinase holoenzyme complex | 5/139 | 42/20758 | 8.73E-06 | 0.000272376 | 0.000203087 | HSPD1/CCNB1/CDK4/CDK1/CCNE2 | 5 |
| GO:0044452 | CC | GO:0044452 | nucleolar part | 9/139 | 219/20758 | 1.72E-05 | 0.000487832 | 0.000363734 | UBE2N/NOL11/POLR2K/RAE1/DKC1/TOP1/TXNRD1/UTP18/NOLC1 | 9 |
| GO:0000779 | CC | GO:0000779 | condensed chromosome, centromeric region | 7/139 | 124/20758 | 2.03E-05 | 0.000526462 | 0.000392537 | NUP107/MAD2L1/CCNB1/AURKA/NCAPG/BIRC5/SPAG5 | 7 |
| GO:0005643 | CC | GO:0005643 | nuclear pore | 6/139 | 85/20758 | 2.29E-05 | 0.000526462 | 0.000392537 | NUP107/XPOT/RAN/MAD2L1/KPNA2/RAE1 | 6 |
| GO:0005759 | CC | GO:0005759 | mitochondrial matrix | 13/139 | 485/20758 | 2.36E-05 | 0.000526462 | 0.000392537 | HSPD1/ATP5F1B/HSPA9/MRPL13/CCNB1/VDAC1/MRPS35/MRPL42/CS/CDK1/MTHFD2/LRPPRC/HSPE1 | 13 |
| GO:0000793 | CC | GO:0000793 | condensed chromosome | 9/139 | 233/20758 | 2.80E-05 | 0.000583376 | 0.000434973 | NUP107/MAD2L1/CCNB1/AURKA/RAD21/NCAPG/BIRC5/SPAG5/LRPPRC | 9 |
| GO:0051233 | CC | GO:0051233 | spindle midzone | 4/139 | 34/20758 | 7.64E-05 | 0.001489589 | 0.001110659 | RACGAP1/AURKA/CDC6/KIF18A | 4 |
| GO:0000922 | CC | GO:0000922 | spindle pole | 7/139 | 158/20758 | 9.54E-05 | 0.001751107 | 0.00130565 | MAD2L1/CCNB1/RAE1/AURKA/CDC6/NPM1/SPAG5 | 7 |
| GO:1902911 | CC | GO:1902911 | protein kinase complex | 6/139 | 117/20758 | 0.000137593 | 0.002384953 | 0.001778254 | HSPD1/CCNB1/CDK4/CDK1/PRKAG1/CCNE2 | 6 |
| GO:0042470 | CC | GO:0042470 | melanosome | 6/139 | 124/20758 | 0.000189088 | 0.002949772 | 0.002199392 | RAN/HSP90AA1/HSP90AB1/YWHAZ/CCT4/YWHAB | 6 |
| GO:0048770 | CC | GO:0048770 | pigment granule | 6/139 | 124/20758 | 0.000189088 | 0.002949772 | 0.002199392 | RAN/HSP90AA1/HSP90AB1/YWHAZ/CCT4/YWHAB | 6 |
| GO:0009295 | CC | GO:0009295 | nucleoid | 4/139 | 44/20758 | 0.000212308 | 0.003010911 | 0.002244977 | ATP5F1B/HSPA9/VDAC1/LRPPRC | 4 |
| GO:0042645 | CC | GO:0042645 | mitochondrial nucleoid | 4/139 | 44/20758 | 0.000212308 | 0.003010911 | 0.002244977 | ATP5F1B/HSPA9/VDAC1/LRPPRC | 4 |
| GO:0000776 | CC | GO:0000776 | kinetochore | 6/139 | 142/20758 | 0.000392736 | 0.005327544 | 0.003972292 | NUP107/MAD2L1/CCNB1/BIRC5/KIF18A/SPAG5 | 6 |
| GO:0005838 | CC | GO:0005838 | proteasome regulatory particle | 3/139 | 22/20758 | 0.000412189 | 0.00535846 | 0.003995343 | PSMD12/PSMD10/PSMD14 | 3 |
| GO:1904813 | CC | GO:1904813 | ficolin-1-rich granule lumen | 6/139 | 146/20758 | 0.000455321 | 0.005682402 | 0.004236879 | CAND1/HSP90AA1/CCT8/HSP90AB1/PSMD12/PSMD14 | 6 |
| GO:0031965 | CC | GO:0031965 | nuclear membrane | 9/139 | 340/20758 | 0.00048465 | 0.005815801 | 0.004336343 | YEATS4/NUP107/MRPS23/CDK4/TMEM97/UTP18/LEMD3/LMNB1/LRPPRC | 9 |
| GO:0034399 | CC | GO:0034399 | nuclear periphery | 6/139 | 151/20758 | 0.000544198 | 0.006288514 | 0.004688804 | YEATS4/NUP107/XPOT/RAD21/PSMA6/LMNB1 | 6 |
| GO:1902554 | CC | GO:1902554 | serine/threonine protein kinase complex | 5/139 | 103/20758 | 0.000648174 | 0.007086282 | 0.005283631 | HSPD1/CCNB1/CDK4/CDK1/CCNE2 | 5 |
| GO:0005876 | CC | GO:0005876 | spindle microtubule | 4/139 | 59/20758 | 0.000658661 | 0.007086282 | 0.005283631 | AURKA/CDK1/KIF18A/SPAG5 | 4 |
| GO:0001650 | CC | GO:0001650 | fibrillar center | 6/139 | 158/20758 | 0.00069065 | 0.007182759 | 0.005355566 | UBE2N/RAE1/DKC1/TOP1/TXNRD1/NOLC1 | 6 |
| GO:0044445 | CC | GO:0044445 | cytosolic part | 8/139 | 287/20758 | 0.000715057 | 0.007196698 | 0.005365959 | CCT2/CCT5/CCT8/CCT4/PSMD14/CCT7/TCP1/CCT6A | 8 |
| GO:0022624 | CC | GO:0022624 | proteasome accessory complex | 3/139 | 27/20758 | 0.000763959 | 0.007222889 | 0.005385488 | PSMD12/PSMD10/PSMD14 | 3 |
| GO:0097431 | CC | GO:0097431 | mitotic spindle pole | 3/139 | 27/20758 | 0.000763959 | 0.007222889 | 0.005385488 | RAE1/AURKA/SPAG5 | 3 |
| GO:0000777 | CC | GO:0000777 | condensed chromosome kinetochore | 5/139 | 111/20758 | 0.000909364 | 0.008344756 | 0.006221967 | NUP107/MAD2L1/CCNB1/BIRC5/SPAG5 | 5 |
| GO:0030496 | CC | GO:0030496 | midbody | 6/139 | 171/20758 | 0.001041873 | 0.009287556 | 0.006924932 | RAN/RACGAP1/AURKA/CDK1/BIRC5/SPAG5 | 6 |
| GO:0061695 | CC | GO:0061695 | transferase complex, transferring phosphorus-containing groups | 8/139 | 308/20758 | 0.001126921 | 0.00976665 | 0.007282151 | HSPD1/CCNB1/PRIM1/POLR2K/CDK4/CDK1/PRKAG1/CCNE2 | 8 |
| GO:0051082 | MF | GO:0051082 | unfolded protein binding | 16/138 | 142/19443 | 4.34E-15 | 1.48E-12 | 1.22E-12 | CCT2/PTGES3/HSPD1/CCT5/HSP90AA1/HSPA9/CCT8/HSP90AB1/HSPH1/CCT4/CCT7/TCP1/NPM1/DNAJA1/CCT6A/HSPE1 | 16 |
| GO:0044183 | MF | GO:0044183 | protein binding involved in protein folding | 10/138 | 54/19443 | 4.28E-12 | 7.27E-10 | 5.99E-10 | CCT2/HSPD1/CCT5/HSPA9/CCT8/HSPH1/CCT4/CCT7/TCP1/CCT6A | 10 |
| GO:0044389 | MF | GO:0044389 | ubiquitin-like protein ligase binding | 16/138 | 377/19443 | 1.13E-08 | 1.29E-06 | 1.06E-06 | CCT2/HSPD1/UBE2N/MDM2/PA2G4/HSPA9/TUBA1B/CCNB1/UBE2V2/AURKA/YWHAZ/DERL1/TCP1/DNAJA1/CACYBP/LRPPRC | 16 |
| GO:0031625 | MF | GO:0031625 | ubiquitin protein ligase binding | 15/138 | 363/19443 | 4.76E-08 | 4.05E-06 | 3.33E-06 | CCT2/HSPD1/UBE2N/MDM2/PA2G4/HSPA9/TUBA1B/UBE2V2/AURKA/YWHAZ/DERL1/TCP1/DNAJA1/CACYBP/LRPPRC | 15 |
| GO:0031072 | MF | GO:0031072 | heat shock protein binding | 8/138 | 150/19443 | 1.18E-05 | 0.000805638 | 0.000663467 | PTGES3/HSPA9/HSP90AB1/STIP1/FKBP4/HSPH1/CDK1/DNAJA1 | 8 |
| GO:0000049 | MF | GO:0000049 | tRNA binding | 5/138 | 58/19443 | 5.67E-05 | 0.002999304 | 0.002470015 | XPOT/METTL1/MARS/RARS/TARS | 5 |
| GO:0015631 | MF | GO:0015631 | tubulin binding | 11/138 | 366/19443 | 6.18E-05 | 0.002999304 | 0.002470015 | CCT5/RACGAP1/RAE1/VBP1/HSPH1/BIRC5/KIF18A/SPAG5/CACYBP/LRPPRC/MAPRE1 | 11 |
| GO:0051087 | MF | GO:0051087 | chaperone binding | 6/138 | 107/19443 | 0.000115128 | 0.004892951 | 0.004029489 | PTGES3/HSPD1/STIP1/BIRC5/DNAJA1/HSPE1 | 6 |
| GO:0097718 | MF | GO:0097718 | disordered domain specific binding | 4/138 | 37/19443 | 0.000133778 | 0.005053817 | 0.004161967 | MDM2/HSP90AA1/HSP90AB1/PPIL1 | 4 |
| GO:0035173 | MF | GO:0035173 | histone kinase activity | 3/138 | 18/19443 | 0.000264035 | 0.0089772 | 0.007392988 | CCNB1/AURKA/CDK1 | 3 |
| GO:0070182 | MF | GO:0070182 | DNA polymerase binding | 3/138 | 19/19443 | 0.000311917 | 0.009641056 | 0.007939693 | PTGES3/HSP90AA1/HSP90AB1 | 3 |
